# A cytidine deaminase-like protein modulates pyrimidine nucleotide homeostasis in *Trypanosoma brucei*

**DOI:** 10.1038/s41598-025-00942-2

**Published:** 2025-05-09

**Authors:** Ana Moro-Bulnes, Cristina Bosch-Navarrete, Pablo Antequera-Parrilla, Santiago Castanys, Antonio E. Vidal, Luis Miguel Ruiz-Pérez, Guiomar Pérez-Moreno, Dolores González-Pacanowska

**Affiliations:** Instituto de Parasitología y Biomedicina “López-Neyra” (IPBLN), CSIC, Parque Tecnológico de Ciencias de la Salud, Avda. del Conocimiento, 17, Armilla, Granada, 18016 Spain

**Keywords:** Pyrimidine metabolism, Cytidine deaminase, *Trypanosoma brucei*, Nucleotide pools, DNA integrity, Stress granules, Enzymes, Parasitology

## Abstract

Pyrimidine nucleotide homeostasis is critical for DNA replication and cell viability, yet its regulation in *Trypanosoma brucei*, the causative agent of African trypanosomiasis, remains poorly understood. Here, we characterize a *T. brucei* cytidine deaminase-like protein (TbCDA-like), a kinetoplastid-specific enzyme absent in mammals, that harbors a deaminase domain and a zinc-finger CCCH motif. Using RNA interference (RNAi) and overexpression approaches, we demonstrate that TbCDA-like modulates pyrimidine nucleotide pools, influencing both ribonucleotide and deoxyribonucleotide profiles. Overexpression of TbCDA-like resulted in a substantial reduction of dCTP and CTP levels while elevating dTTP and UTP pools, suggesting a role in cytidine derivative deamination. Conversely, RNAi-mediated depletion of TbCDA-like caused CMP accumulation and reduced dTTP levels. Notably, overexpression induced severe cytotoxicity, growth arrest, DNA damage and cell cycle defects, evidenced by sub-G1 populations, increased nuclear H2A phosphorylation and aberrant kinetoplast and nuclear morphologies. Localization studies revealed that TbCDA-like is primarily cytosolic and relocalizes to stress granules upon nutrient deprivation, suggesting a role in RNA metabolism. These findings establish TbCDA-like as a key regulator of pyrimidine nucleotide homeostasis in *T. brucei*, linking nucleotide imbalances to DNA replication stress and genome instability. Given its absence in mammals, TbCDA-like presents an attractive target for therapeutic intervention against trypanosomiasis.

**Supplementary Information:**

The online version contains supplementary material available at 10.1038/s41598-025-00942-2.

## Introduction

The extracellular protozoan parasite *Trypanosoma brucei* is the etiological agent of African trypanosomiasis. *T. brucei* comprises different subspecies, with *T. brucei rhodesiense* and *T. brucei gambiense* responsible for human African trypanosomiasis (HAT), commonly known as sleeping sickness^1^, while *T. brucei brucei* primarily affects wild and domestic animals^2^. Despite considerable research into the biology of this parasite, many aspects of nucleotide metabolism, DNA repair and their underlying mechanisms remain poorly understood^3–6^.

Pyrimidines play a crucial role in the structure and function of nucleic acids, providing both structural integrity and genetic information. Maintaining appropriate deoxyribonucleotide (dNTP) homeostasis is essential for DNA biosynthesis, replication, and cell proliferation^7^. The degradation and salvage of dNTPs involve a range of enzymes, including 5’-nucleotidases, nucleoside phosphorylases, kinases and deaminases. While the regulation of dNTPs throughout the cell cycle is well-documented in mammalian cells, knowledge regarding this process in trypanosomes is still limited^7^.

In our quest to identify genes encoding nucleotide-metabolizing enzymes within the *T. brucei* genome, we focused on members of the deaminase superfamily, enzymes known to catalyze the deamination of bases in nucleotides and nucleic acids under various biological conditions. This superfamily includes cytidine deaminases (CDD/CDA), deoxycytidylate monophosphate deaminases (DCTD) and guanine deaminase (GuaD), which are primarily involved in the salvage and catabolism of pyrimidines and purines^8^. Some of these deaminases are also capable of catalyzing in situ base deamination in both RNA and DNA. In *T. brucei*, Tb927.10.8850 was initially identified as a member of a unique branch within the CDD/CDA-like clade^8^, which we refer to here as TbCDA-like. The presence of a zinc-finger nucleic acid-binding motif in this enzyme suggests potential DNA or RNA binding activity, though its involvement in pyrimidine salvage cannot be ruled out. The functional consequences of DNA or RNA binding are unclear yet may include regulation of transcript accumulation, direct deamination of nucleic acids as substrates or modulation of enzyme activity.

This group of proteins is widely distributed across various microbial eukaryotes, including kinetoplastids, stramenopiles, chlorophyte algae and the alveolate *Perkinsus sp.*, yet no orthologues are apparent in mammals. The kinetoplastid orthologue, first identified in *Leishmania major* (LmjF.36.5940), is considered the representative member of this group, which has been designated as the LmjF.36.5940-like clade^8^.

In the present study, we explore the potential function of the TbCDA-like enzyme in *T. brucei brucei* procyclic cells. Our primary objective was to elucidate its role in pyrimidine nucleotide homeostasis in the parasite. Our findings demonstrate that modulation of enzyme levels leads to alterations in both dNTP and rNTP pools. Moreover, we show that overexpression of TbCDA-like impacts genomic integrity and disrupts cell cycle progression, likely due to perturbations in nucleotide pool equilibrium.

## Results

### *T. brucei* encodes a cytidine deaminase-like of unknown function

BLAST-P searches using the LmjF.36.5940 (CDA-like) sequence from *Leishmania major* as a query identified orthologues in the class Kinetoplastea, exhibiting conserved gene synteny. The TbCDA-like protein is a 65.89 kDa enzyme composed of 585 amino acids, with a calculated pI of 7.13, encoded by a single-copy gene located on chromosome 10.

An alignment was performed with four CDA-like sequences: *T. brucei* CDA-like, *L. major* CDA-like (representative of the group), *T. cruzi* CDA-like and *C. fasciculata* CDA-like (Supplementary Fig. S1). Kinetoplastid CDA-like is characterized by the presence of a CCCH zinc finger motif^9^, a feature commonly associated with proteins that interact with nucleic acids, as well as a deaminase domain (Supplementary Fig. S1).

Apart from the CCCH motif, described as C-X_5_-C-X_5_-C-X_3_-His, which is a non-conventional zinc finger domain^9^, cysteines that could constitute a C2C2 domain possibly involved in zinc coordination^8^ are also conserved. Finally, as suggested by Iyer et al., the presence of an additional CCCH motif may be considered^8^. Specifically, the cysteine residues Cys134, Cys143, and Cys145, along with histidine His149—conserved in all analyzed orthologues—define a region that may represent another non-conventional CCCH motif (C-X_9_-C-X_2_-C-X_3_-H), potentially involved in nucleic acid-binding activity (Supplementary Fig. S1).

The deaminase domain, characterized by the consensus sequence C-X-E-X_24 − 36_-P-C-X_2_-C^9^, is located at the C-terminus and is conserved across all species analyzed, including *T. brucei* (Cys483, Glu485, Pro523, Cys524, and Cys527). Supplementary Fig. S2 presents an alignment of two cytidine deaminase protein sequences (HsCDA and TbCDA), along with the deaminase domain-containing regions of TbCDA-like and LmCDA-like and reveals that the amino acids critical for the deaminase activity are all present in kinetoplastid CDA-like proteins.

### Modulation of the expression of TbCDA-like results in perturbed nucleotide pools

**Fig. 1:**
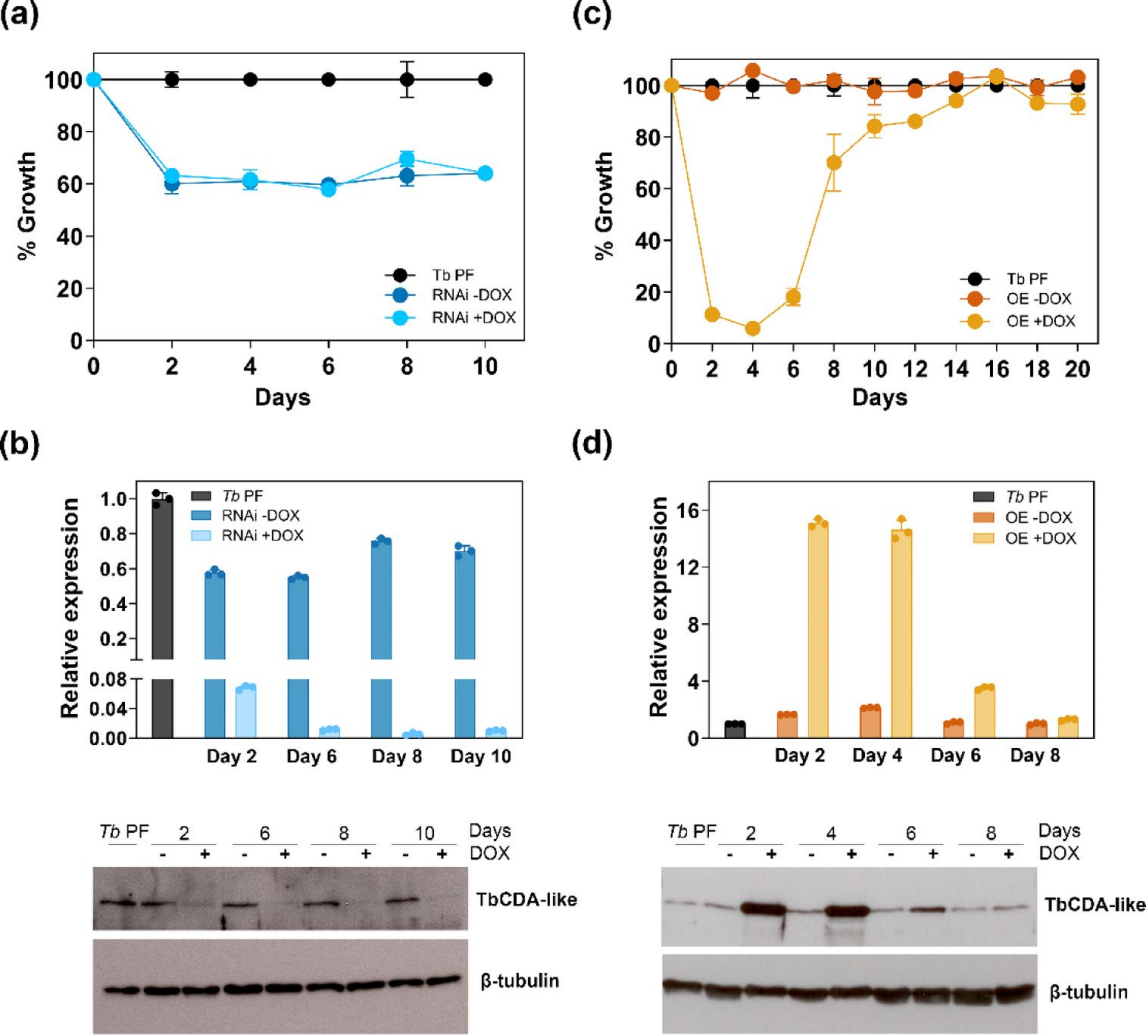
Impact of TbCDA-like modulation on growth and protein expression in *T. brucei*. (**a**) Growth curves of *Tb* PF and *Tb* PF CDA-like-RNAi cell lines. DOX, doxycycline. Each point represents the mean from three biological replicates. (**b**) Western blot quantification demonstrating efficient reduction of TbCDA-like levels following DOX induction. (**c**) Growth curves of *Tb* PF and the *Tb* PF CDA-like-OE cell lines with or without DOX. Data point represents the mean from three biological replicates. (**d**) Western blot quantification of TbCDA-like expression showing the increase in TbCDA-like expression upon DOX induction. 5 × 10^6^ parasites were loaded in each lane. Anti-TbCDA-like (1:2500) and anti-β-tubulin (1:5000) antibodies were used for detection and normalization, respectively. Protein quantification was performed using Fiji software. Samples and controls derived from the same experiment and blots were processed in parallel. Only the lines of interest are shown from full-length blots. Original blots are presented in Supplementary Fig. S4.

To investigate the biological function of TbCDA-like and its role in the regulation of pyrimidine nucleotide pools, we established a *Tb* PF cell line with a tetracycline-controlled inducible RNA interference (RNAi) system for silencing TbCDA-like expression (*Tb* PF CDA-like-RNAi) (Supplementary Fig. S3). Initially, we compared the proliferation rates of this cell line before RNAi induction with that of the parental strain. We found that uninduced cells showed a reduced cell density compared to *Tb* PF strain cells (Fig. 1a) together with a moderate reduction in TbCDA-like levels suggesting a leaky RNAi phenotype (Fig. 1b). A total of three clones were analyzed; all exhibited the same phenotype. On the other hand, RNAi-mediated depletion did not induce any further detectable changes in cell density compared to uninduced cells. Protein levels during RNAi induction dropped strongly at 2 days post-induction and remained low through 10 days post-induction (Fig. 1b). Growth after RNAi induction was additionally analysed for 10 days in the presence of thymidine (dThd), uracil (Ura), deoxyuridine (dUrd) or uridine (Urd) (Supplementary Fig. S5). However, supplementation with different nucleosides or nucleobases failed to revert the loss-of-viability phenotype. We also generated a *Tb* PF cell line with a tetracycline-controlled inducible expression system to overexpress TbCDA-like (*Tb* PF CDA-like-OE) (Supplementary Fig. S3). Western blot analysis revealed that TbCDA-like levels increased approximately 15-fold after 2 and 4 days of induction (Fig. 1d), but declined thereafter. Overexpression of TbCDA-like resulted in significant cytotoxicity (four independent clones were analyzed), with up to 90% growth inhibition compared to the *Tb* PF cell line at day 4 post-induction. Both protein overexpression and reduced cell proliferation diminished over time, with normal growth being restored after 10 days, further supporting the notion that excessive TbCDA-like production leads to a defective growth phenotype (Fig. 1c, d).

**Fig. 2:**
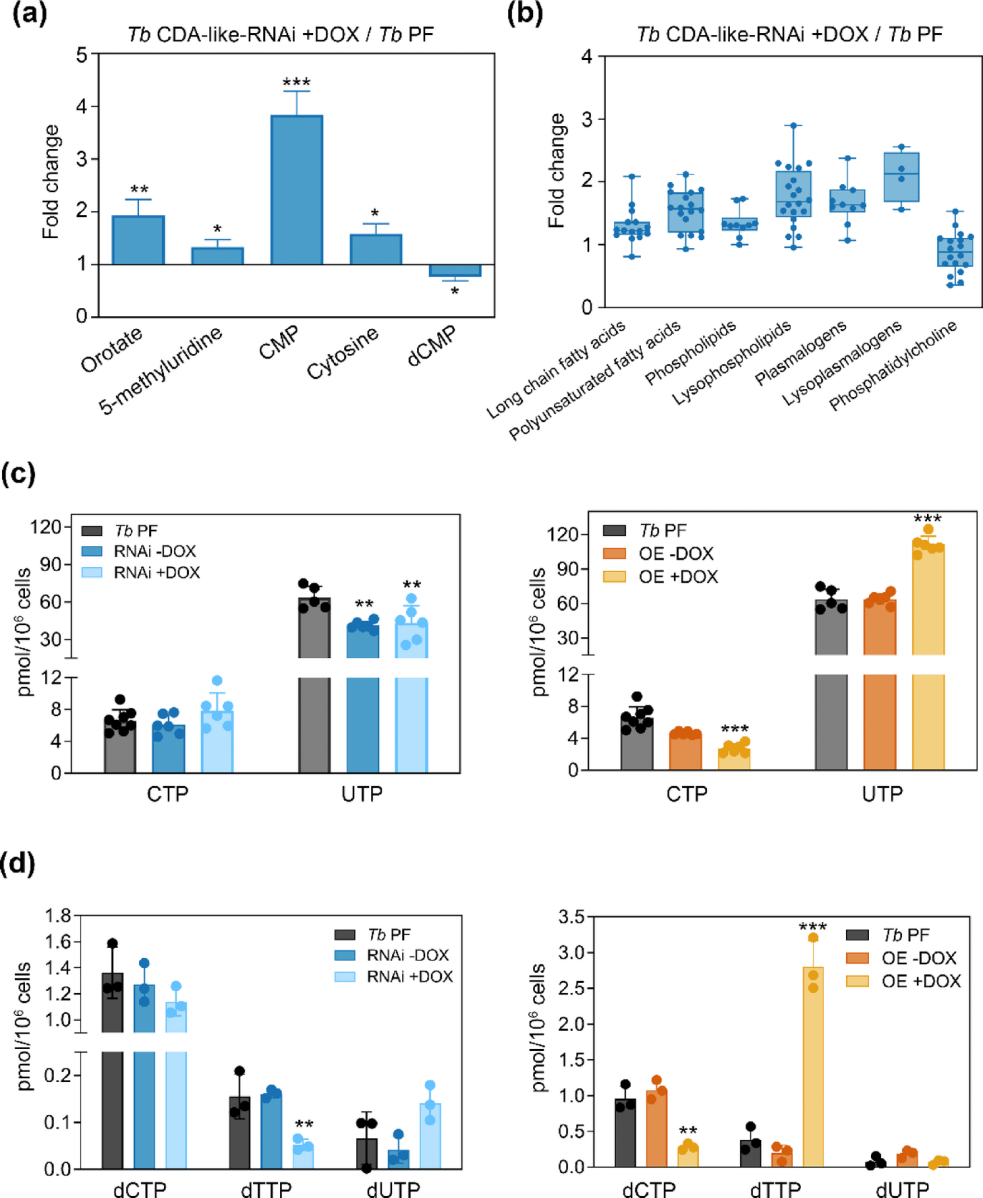
Comparative analysis of metabolite and nucleotide alterations in *Tb* PF CDA-like-RNAi and OE cell lines. (**a**) Relative expression of key metabolites altered in the *Tb* PF CDA-like-RNAi line after 6 days of DOX induction compared to the parental *Tb* PF line, measured using various mass spectrometry techniques. Data are presented as mean ± SD from 4 independent biological replicates. Statistical significance was determined using Welch’s t-test (*p < 0.05, **p < 0.01, ***p < 0.001). (**b**) Relative abundance of different lipid classes in the TbCDA-like-RNAi line compared to the parental *Tb* PF line. Data are presented as mean ± SD from 4 independent biological replicates. Boxes represent interquartile ranges and whiskers denote maximum and minimum values. (**c**) Levels of rNTPs in *Tb* PF, *Tb* PF CDA-like-RNAi and *Tb* PF CDA-like-OE lines, measured by anion exchange chromatography after 2 days of DOX induction. (**d**) Pyrimidine dNTP levels measured by the polymerase-based assay in *Tb* PF CDA-like-RNAi and *Tb* PF CDA-like-OE lines, 4 and 2 days post-induction, respectively. Panels c and d present data as mean concentrations with error bars indicating ± SD from three independent experiments with triplicate technical replicates. Asterisks indicate significant differences calculated by ANOVA. **p < 0.01; ***p < 0.001. The statistical significance analyses were performed by comparing the RNAi + DOX and OE + DOX cell lines with the *Tb* PF cell line.

A global metabolomic analysis of *Tb* PF and *Tb* PF CDA-like-RNAi cell lines was conducted. A total of 565 different metabolites were identified by mass spectrometry, 109 of which were significantly altered in *Tb* PF CDA-like-RNAi after 6 days of induction, compared to the parental cell line (Supplementary Spreadsheet S1). Some of these metabolites are related to pyrimidine metabolism, such as orotate (1.9 fold increase), 5-methyluridine (1.3 fold increase), cytosine (1.6 fold increase) and outstandingly, CMP (3.8 fold increase) (Fig. 2a). In addition, significant changes were observed in various lipid classes following 6 days of TbCDA-like RNAi induction, including long chain fatty acids, polyunsaturated fatty acids, phospholipids, lysophospholipids, plasmalogens, lysoplasmalogens and phosphatidylcholine (Fig. 2b). Increased cytosolic lipid synthesis has been associated with stress responses and cellular apoptosis^10,11^, and the observed lipid accumulation may reflect the cellular adaptation to the phenotype induced by TbCDA-like deficiency.

We next performed a comprehensive analysis of ribonucleoside triphosphates (rNTPs). For this purpose, rNTPs were measured by anion exchange chromatography in the different cell lines generated: *Tb* PF, *Tb* PF CDA-like-OE and *Tb* PF CDA-like-RNAi. After 48 h of RNAi induction, we observed that CTP levels were slightly yet not significantly increased while the UTP pool was moderately diminished however in both induced and uninduced cells thus denoting a leaky RNAi phenotype (Fig. 2c). In contrast, in *Tb* PF CDA-like-OE cells, the CTP pool was substantially reduced and UTP levels significantly expanded upon induction (Fig. 2c). Pyrimidine deoxynucleoside triphosphates (dNTPs) were quantified using the highly sensitive polymerase-based dNTP quantification assay^12^. RNAi-mediated depletion of CDA-like after both 4 days of induction resulted in decreased dTTP levels (Fig. 2d). In *Tb* PF CDA-like-OE cells, however, dCTP levels were significantly reduced, accompanied by a marked increase in dTTP levels (Fig. 2d). Hence, taken together, the results point towards a role of TbCDA-like in pyrimidine nucleotide homeostasis.

### Overexpression of TbCDA-like is cytotoxic and impairs cell cycle progression

**Fig. 3:**
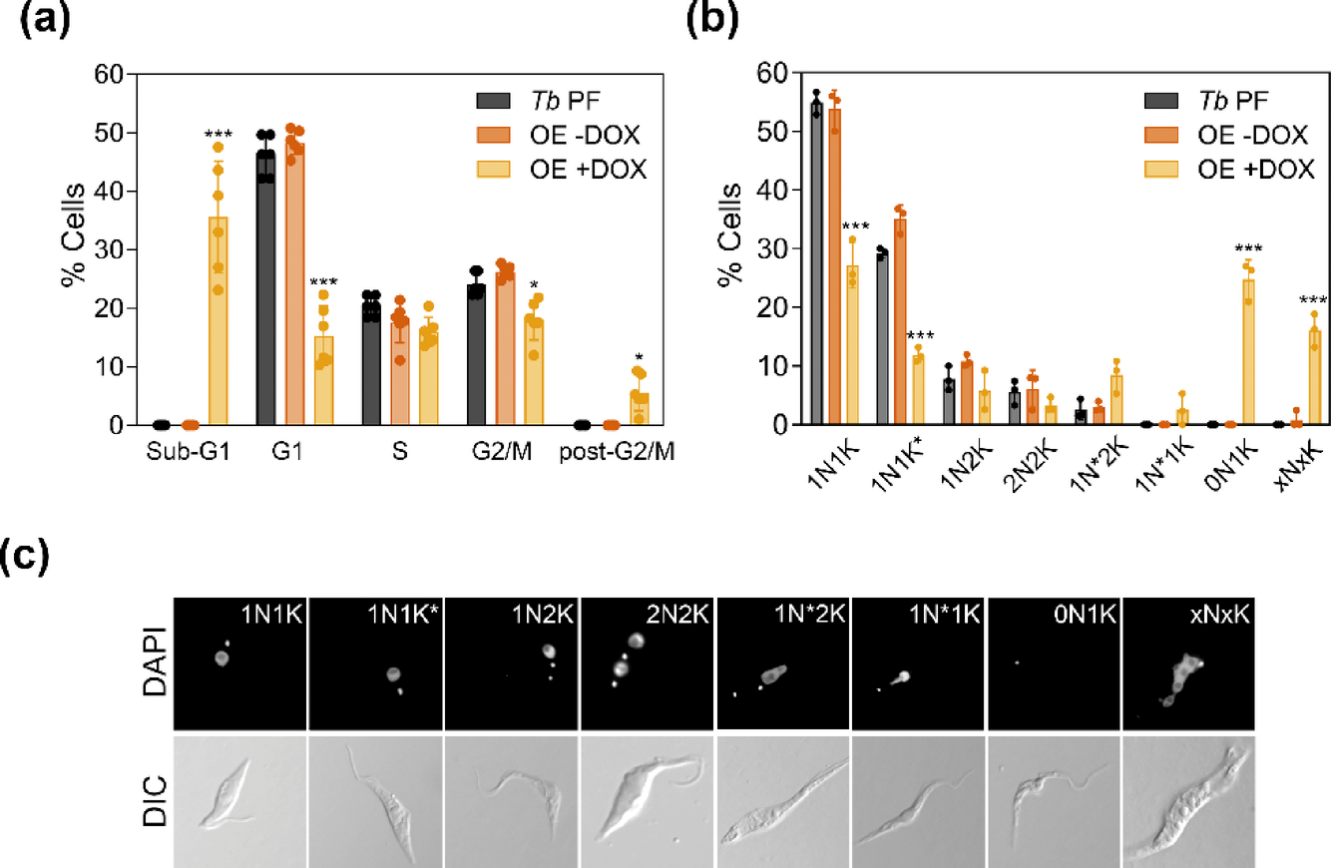
Altered cell cycle progression in *T. brucei* upon TbCDA-like overexpression. (**a**) Distribution of cells across different cell cycle phases after 2 days of overexpression induction, followed by propidium iodide staining and flow cytometry (FACS) analysis. Data are presented as mean ± SD from 3 independent experiments. (**b**) Quantification of nuclei (N) and kinetoplasts (K) after 2 days of overexpression induction, followed by DAPI staining and fluorescence microscopy. N* and K* denote elongated nuclei and kinetoplasts, respectively, indicative of mitosis or kinetoplast division. Data are presented as mean ± SD from approximately 300 cells from 3 independent experiments. Asterisks indicate significant differences determined by ANOVA. *p < 0.05; ***p < 0.001. The statistical significance analyse was performed by comparing the OE + DOX cell line with the *Tb* PF cell line. (**c**) Representative fluorescence microscopy images (Zeiss Axio Imager A1) after DAPI staining, illustrating the different cell populations.

Nucleotide demands fluctuate during cell cycle progression, and disruptions in nucleotide levels at the onset of S phase can activate replication stress signaling, ultimately affecting cell growth and proliferation^13^. To assess whether the loss or overexpression of TbCDA-like influences cell cycle progression, we performed flow cytometry (FACS) analysis and measured nucleus/kinetoplast patterns. The analysis enabled the quantification of cells in the G1/early S phase, late S/G2 phase, mitosis and atypical cell forms, as previously described^14^. DNA content was evaluated by FACS after 2 days of RNAi induction or overexpression of TbCDA-like. Representative flow cytometry histograms for cell cycle analysis of *Tb* PF and *Tb* PF CDA-like-RNAi and *Tb* PF CDA-like-OE cell lines are shown in Supplementary Fig. S6. After 2 days of TbCDA-like overexpression, we observed a significant increase in the proportion of sub-G1 cells (35.6%), accompanied by a decrease in G1 and G2/M populations (15.3% and 18% respectively) (Fig. 3a). In addition, an increase of 5.6% of polyploid cells (post-G2/M) is observed. The sub-G1 peak represents cells with degraded nuclear DNA, premature DNA replication termination or unscheduled mitosis as well as anucleate parasites containing only kDNA. DAPI staining to examine nuclei and kinetoplast morphology revealed that after 2 days of induction, in *Tb* PF CDA-like-OE cells there was a decrease in 1N1K and 1N1K*(27.2% and 11.9% versus 54.9% and 29.3% in control cells, respectively) populations (corresponding to G1 phase), an increase in 0N1K cells (24.8% versus 0%) known as zoids, as well as a significant increase in XNXK cells (16.1% versus 0%) that exhibit an abnormal number and/or morphology of nuclei and kinetoplasts. Additionally, an increase in the 1N*2K (8.5%) and 1N*1K (2.5%) populations (Fig. 3b, c) compared to the control cell line (2.5% and 0%, respectively) were also detected.

RNAi-mediated depletion of TbCDA-like did not cause significant cell cycle perturbations (Supplementary Fig. S7). However, a minor reduction in 1N1K population and an increase in abnormal populations, not detectable in parental *Tb* PF cells, were identified by DAPI staining: XNXK (3.3%) and 0N1K (2.1%) (Supplementary Fig. S7). These changes were evident in both induced and uninduced cells, likely reflecting the leaky RNAi phenotype.

### TbCDA-like overexpression results in γH2A accumulation

**Fig. 4:**
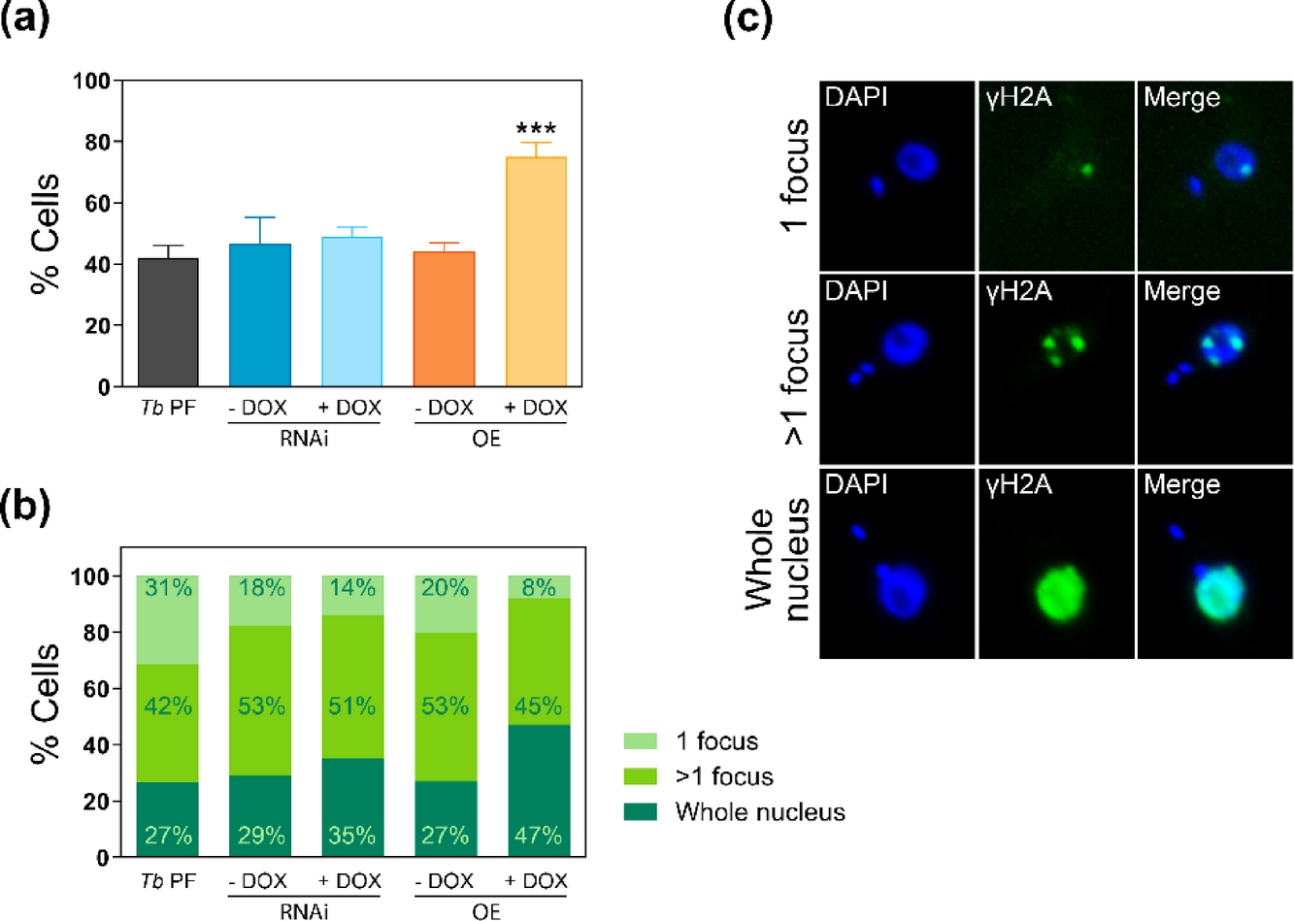
Modulation of TbCDA-like expression induces DNA damage and alters the distribution of nuclear foci in *T. brucei* cell lines. (**a**) Percentage of γH2A-positive cells in *Tb* PF, *Tb* CDA-like-RNAi and *Tb* CDA-like-OE cell lines after 2 days of induction. Data are presented as mean ± SD of approximately 250 cells from 3 independent experiments. (**b**) Distribution of cells displaying 1 focus, > 1 focus (2–4 foci) or whole nucleus labeling for γH2A after 2 days of induction. (**c**) Representative immunofluorescence images showing nuclei labeled for γH2A, with 1 focus, > 1 focus or the whole nucleus. Images were captured using a Zeiss Axio Imager A1 fluorescence microscope following DAPI staining and immunolabeling with a polyclonal anti-γH2A antibody, followed by an anti-rabbit IgG conjugated to Alexa Fluor 488. Asterisks indicate significant differences determined by ANOVA. ***p < 0.001. The statistical significance analysis was performed by comparing the OE + DOX cell line with the *Tb* PF cell line.

Imbalance in nucleotide pools are known to result in DNA damage^15^ and the fidelity of DNA synthesis and repair is highly dependent on a balanced pool of dNTPs. The emergence of a sub-G1 population prompted us to investigate whether the perturbation of nucleotide pools resulting from modulation of TbCDA-like expression affects the DNA damage response. To address this, we quantified the phosphorylation of Thr130 of trypanosomal histone H2A, known as γH2A, which is a key early event in the DNA damage response and plays a critical role in double-strand break repair^16^. Immunofluorescence analysis using the anti-TbγH2A antibody was performed on *Tb* PF, *Tb* PF CDA-like-OE and *Tb* PF CDA-like-RNAi cell lines. Examination of approximately 300 cells per condition revealed that a significant increase in the number of cells with nuclear γH2A staining, compared to the parental cell line, was observed in *Tb* PF CDA-like-OE cells after 48 h of induction, with 32.9% more cells exhibiting γH2A staining compared to the parental *Tb* PF line (Fig. 4a). In contrast, RNAi induction did not induce any significant change in γH2A levels (Fig. 4a). In addition, the distribution of γH2A labeling after 2 days of induction was analyzed and it was found that in *Tb* PF CDA-like-OE cells, the number of cells with the whole nucleus staining increased by 20% compared to the parental *Tb* PF line, while the number of cells with a single focus decreased by 23% (Fig. 4b). Figure 4c illustrates the different γH2A labeling patterns considered in this study.

### TbCDA-like intracellular localization

**Fig. 5:**
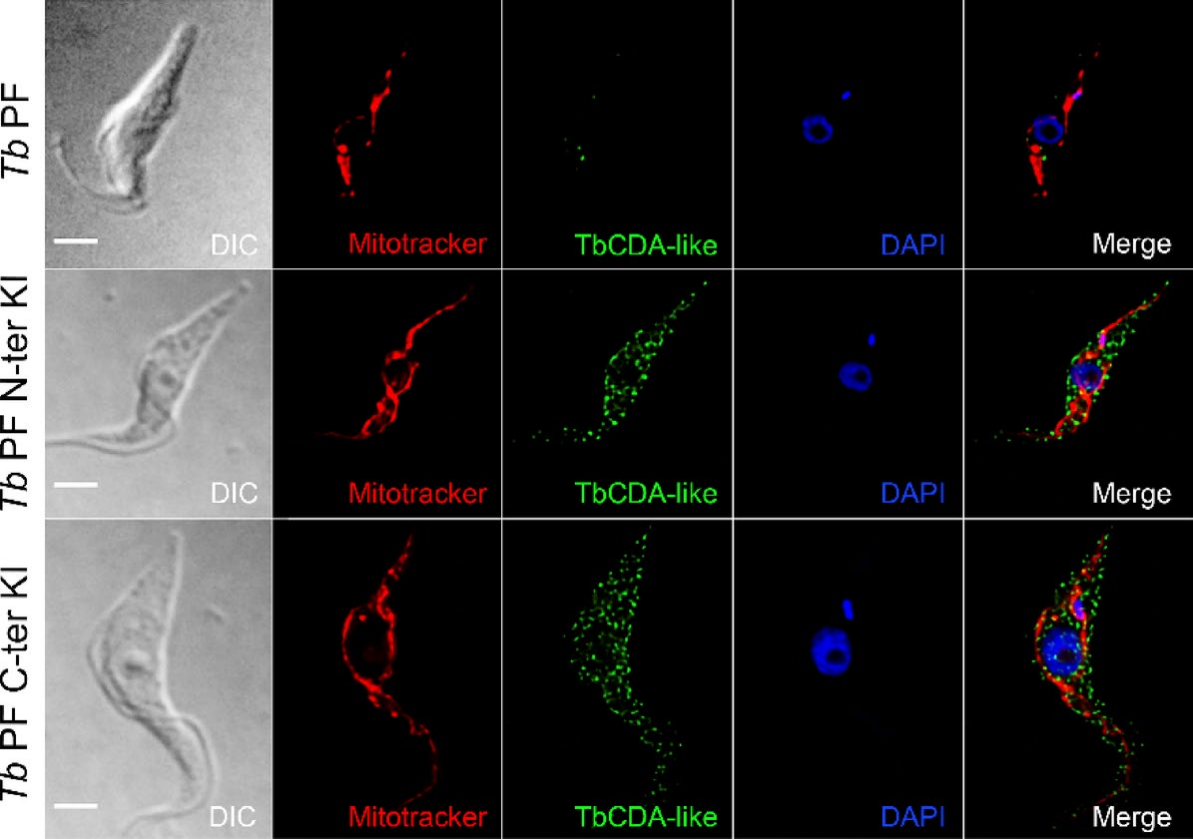
TbCDA-like is localized in the cytosol. Localization of TbCDA-like fused to 6x c-myc at the N-terminus in *Tb* PF N-ter KI cells and of TbCDA-like fused to 12x c-myc at the C-terminus in the *Tb* PF C-ter KI cell line. The parental *Tb* PF line was used as a negative control. Immunolabeling was performed with a mouse monoclonal anti-c-myc antibody and Alexa Fluor 488-conjugated anti-mouse IgG. Mitochondria were stained with MitoTracker Red CMXRos, and the nucleus and kinetoplast were visualized using DAPI. Images were captured using an Olympus IX81 microscope and deconvoluted using the Huygens Essential software (version 3.3; Scientific Volume Imaging). Images were analyzed with Fiji/ImageJ software. Bar, 10 μm.

To investigate the biological function of TbCDA-like, we examined its intracellular localization. For this purpose, two knock-in (KI) cell lines were generated which express TbCDA-like fused to 6x or 12x c-myc tags in the N or C-terminal region, respectively (Supplementary Fig. S3). These cell lines were used for immunofluorescence analysis with an anti-c-myc antibody (Western blot validation of antibody specificity is shown in Supplementary Fig. S8). Examination of *Tb* PF KI cells expressing TbCDA-like fused to 6x c-myc tags in the N-terminus (*Tb* PF N-ter KI) and *Tb* PF KI cells fused to 12x c-myc tags in the C-terminus (*Tb* PF C-ter KI) illustrates that TbCDA-like exhibits a irregular granular staining pattern and localizes predominantly in the cytosol (Fig. 5). Immunofluorescence analysis was also performed in wild-type and overexpressing cells using the anti-TbCDA-like antibody (Supplementary Fig. S9). When overexpressed, TbCDA-like accumulates in the nuclear periphery. Interestingly, this is a characteristic feature of some RNA binding proteins (RBPs)^17^. A potential glycosomal localization was excluded based on colocalization analysis with mevalonate kinase^18^, which yielded a Pearson’s coefficient of 0.26 (Supplementary Fig. S10).

### TbCDA-like is present in stress granules after starvation

**Fig. 6:**
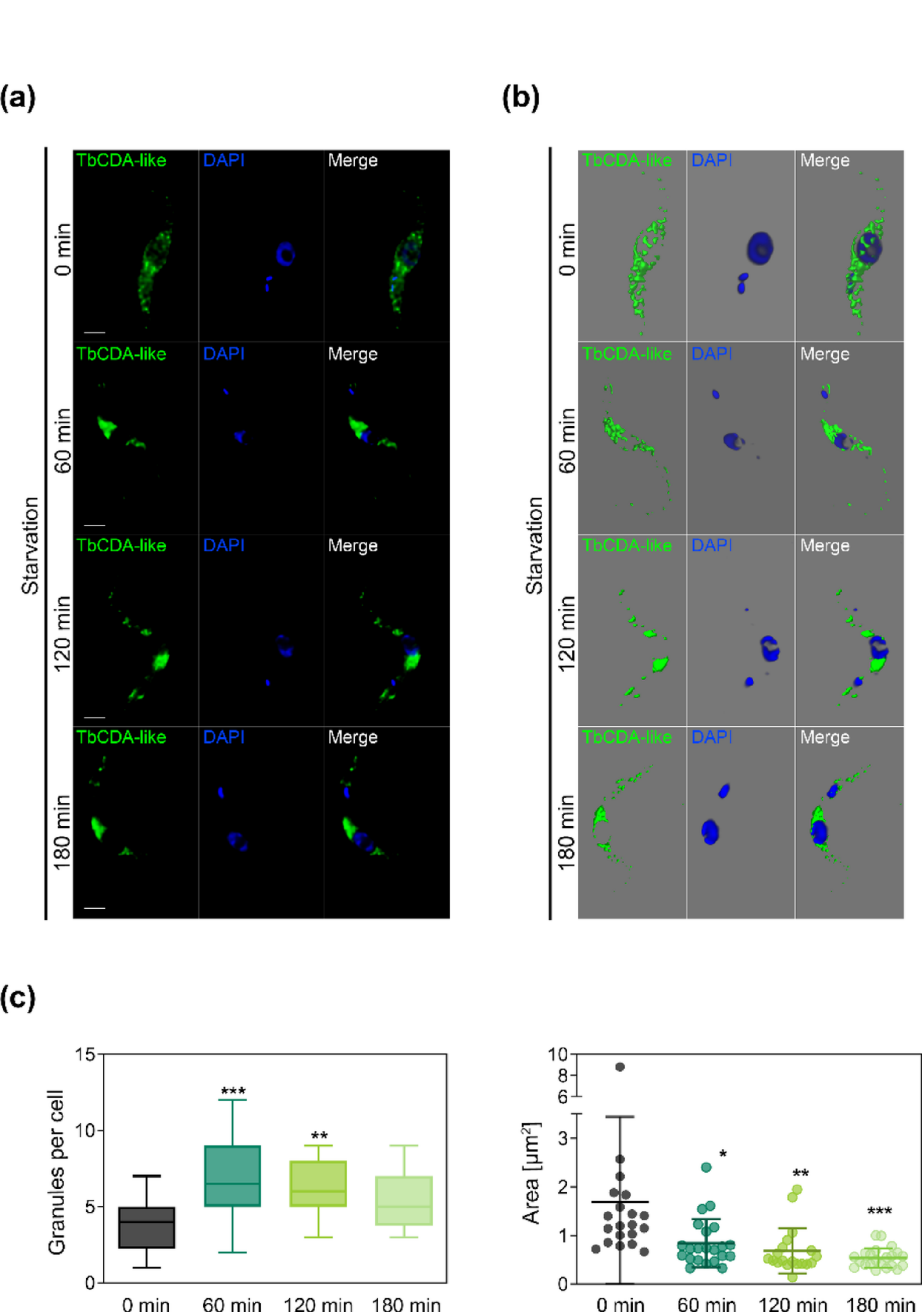
TbCDA-like relocalizes to perinuclear granules under starvation conditions, with an increase in number and a decrease in size compared to untreated cells. (**a**) Immunofluorescence images showing the distribution of TbCDA-like in the TbCDA-like KI line with 6x c-myc fusion at the N-terminus (*Tb* PF N-ter KI) under normal (0 min control) and starvation conditions for 60, 120, or 180 min. Cells were stained with anti-c-myc monoclonal antibody and Alexa Fluor 488-conjugated anti-mouse IgG secondary antibody. Nuclei and kinetoplasts were labeled with DAPI. Scale bar, 10 μm. (**b**) 3D construction of images obtained with the three-dimensional reconstruction module of Leica Application Suite X software. (**c**) Quantification of the number of granules per cell, with a minimum granule area of 0.05 μm^2^. Data represent the mean of at least 20 cells per condition, with boxes showing the interquartile range and whiskers indicating the maximum and minimum values. (**d**) Analysis of the granule area per cell, with a minimum area of 0.05 μm^2^. Data are presented as the mean ± SD for 20–22 cells per condition. Statistical significance was determined by ANOVA (*p < 0.05, **p < 0.01, ***p < 0.001). Images were analyzed using Fiji/ImageJ software.

To further investigate the potential association of TbCDA-like with RNA granules, particularly stress granules (SGs), we employed the *Tb* PF N-ter KI cell line and performed immunofluorescence analysis using an anti-c-myc antibody, following starvation-induced stress by culturing the cells in PBS for varying durations. The images presented in Fig. 6a illustrate the time course for serum starvation. Figure 6b shows the 3D construction of immunofluorescence images. An analysis with Fiji/ImageJ software establishing a minimum particle size of 0.05 µm^2^, indicates that upon serum starvation TbCDA-like locates to granules, which increase in number and decrease in size after 60, 120 and 180 min (Fig. 6c).

**Fig. 7:**
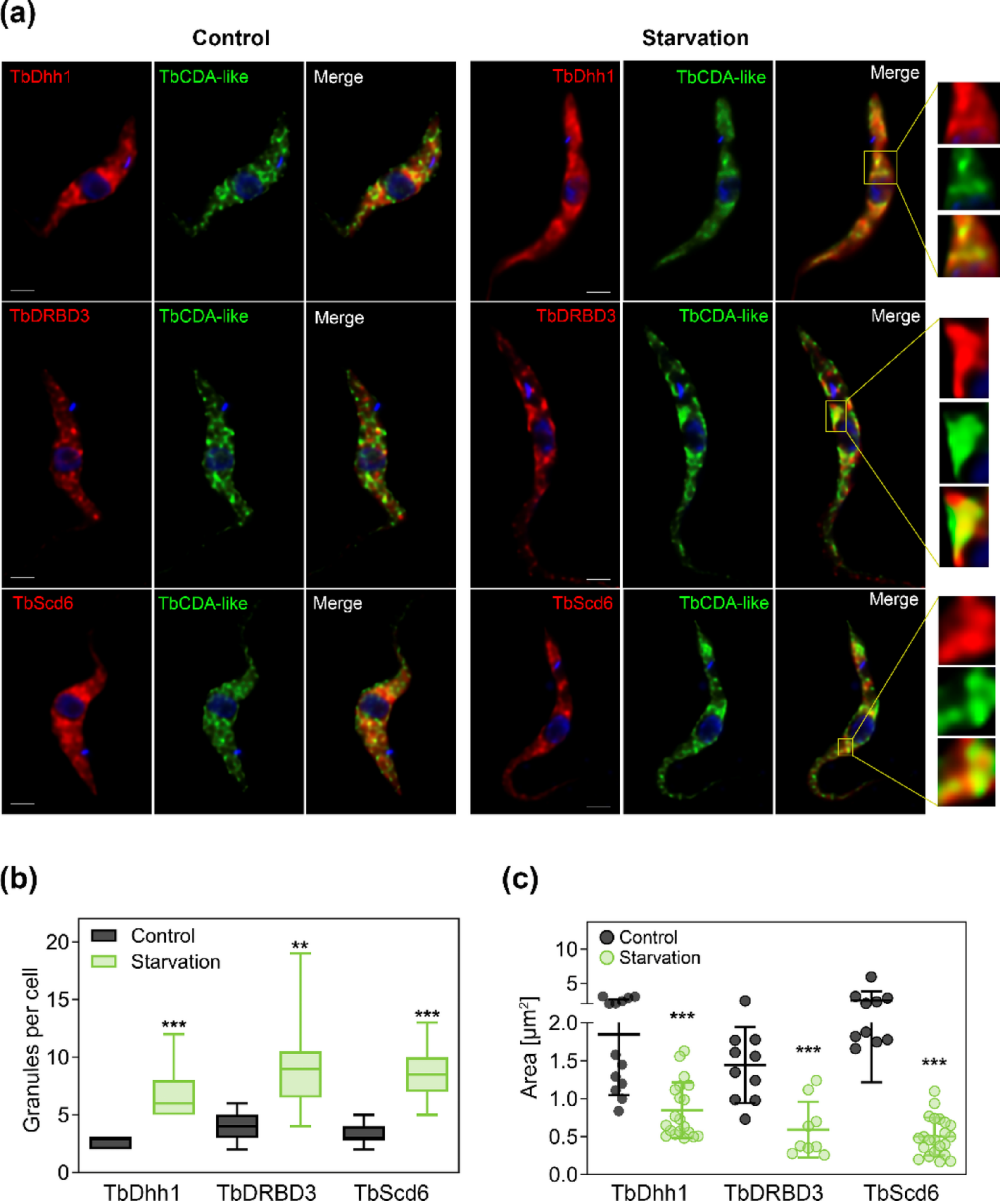
TbCDA-like colocalizes with stress granule marker proteins in *T. brucei*. (**a**) Colocalization of TbCDA-like with stress granules marker proteins TbDhh1, TbDRBD3 and TbScd6 under normal (control) and starvation conditions (120 min) in the TbCDA-like KI line with 6x c-myc fusion at the N-terminus. An anti-c-myc monoclonal antibody and the corresponding anti-Dhh1, anti-DRBD3 or anti-Scd6 polyclonal antibodies were used as primary antibodies. Alexa Fluor 488-conjugated anti-mouse IgG and Alexa Fluor 594-conjugated anti-rabbit IgG were used as secondary antibodies. Nuclei and kinetoplasts were labelled with DAPI. Images were acquired using a Leica DMi8 microscope and deconvoluted with Huygens Essential software (version 3.3; Scientific Volume Imaging). Bar, 10 μm. (**b**) Quantification of granules per cell with a minimum area of 0.05 μm^2^. Data represent the mean from at least 10 cells per condition, with boxes indicating the interquartile range and whiskers showing the maximum and minimum values. (**c**) Granule area per cell analysis, considering a minimum granule area of 0.05 μm^2^. Data are presented as mean ± SD from 10–22 cells per condition. Statistical significance was determined by ANOVA (*p < 0.05, **p < 0.01, ***p < 0.001). Images were analyzed using Fiji/ImageJ software.

To confirm the identity of these structures as SGs, we conducted colocalization analysis of TbCDA-like with the established SG markers TbDhh1^19^, TbDRBD3^20^ and TbScd6^21^ under starvation conditions (Fig. 7). Immunofluorescence was performed using an anti-c-myc monoclonal antibody in combination with polyclonal anti-TbDhh1 (top panels), anti-TbDRBD3 (middle panels), or anti-TbScd6 (bottom panels) (Fig. 7a). Upon 120 min starvation, the number of granules per cell for each of these SG proteins was significantly higher (Fig. 7b) while their area was smaller compared to control cells (Fig. 7c). Importantly, we found significant colocalization of TbCDA-like with all three SG markers after 120 min of starvation. Thus, quantitative analysis was performed by calculating the Pearson’s coefficient and the data obtained correspond to the mean (± SD) of 10–20 cells. Under standard growth conditions, colocalization coefficients for TbCDA-like were 0.82 ± 0.03 for TbDhh1, 0.84 ± 0.03 for TbDRBD3 and 0.81 ± 0.06 for TbScd6. Similar values were obtained upon 120 min of starvation (0.8 ± 0.05 with TbDhh1, 0.78 ± 0.05 with TbDRBD3, and 0.78 ± 0.04 with TbScd6).

## Discussion

In this study, we sought to elucidate the biological function of TbCDA-like, a unique enzyme representative of a divergent branch within the CDD/CDA-like clade in kinetoplastids. Sequence analysis suggested that TbCDA-like might be involved in free nucleotide metabolism, while the presence of a zinc-finger nucleic acid-binding motif raised the possibility of its involvement in RNA or DNA binding. While deaminases that act on tRNAs (e.g., ADATs) are well-characterized in *Trypanosoma brucei*^22,23^, to date, no deaminases acting on mRNA have been reported, nor is there evidence for mRNA editing through cytosine/adenine deamination in these organisms.

A recent analysis by Paris et al.^24^ identified proteins in *T. brucei* containing both deaminase (C/H-X-E) and zinc-finger (P-C-X-X-C) motifs, highlighting 116 proteins with potential roles in nucleotide metabolism. Among these, 17 proteins were predicted to localize to the mitochondrion and carried both motifs, including Tb927.10.8850 (TbCDA-like). However, our detailed intracellular localization analysis, supplemented by data from TrypTag (TrypTag.org), clearly showed that TbCDA-like is predominantly cytosolic rather than mitochondrial, indicating a distinct functional role from mitochondrial RNA editing or tRNA deamination. Furthermore, the absence of nuclear localization also argues against a role in DNA deamination, which has not been described in trypanosomatids to date. Additionally, given that key tRNA deaminases have already been characterized in *T. brucei*^22,23,25^, it is unlikely that TbCDA-like is involved in tRNA deamination. The absence of TbCDA-like in the mitochondrion further excludes it from participating in mitochondrial tRNA deamination processes.

Our findings underscore the significant role of TbCDA-like in pyrimidine metabolism, specifically in regulating pyrimidine ribonucleotide triphosphates (rNTPs) and deoxyribonucleotide triphosphates (dNTPs). Modulation of TbCDA-like expression revealed that its overexpression leads to a reduction in cytidine triphosphate (CTP) levels and an accumulation of uridine triphosphate (UTP), while RNAi-mediated depletion results in a modest but significant decrease in UTP levels. The leaky phenotype observed in uninduced cells must be considered when interpreting these data. Moreover, our analysis of pyrimidine dNTPs revealed changes in both dCTP and particularly dTTP pools, indicating that TbCDA-like levels directly impact pyrimidine homeostasis. The substantial reduction in CTP and dCTP levels upon TbCDA-like overexpression may be attributed to increased deamination, depleting the cell of cytidine and/or deoxycytidine intermediates. Given that direct cytosine or cytidine salvage is virtually absent in *T. brucei*, compensation must rely on *de novo* synthesis pathways involving CTP synthetase and ribonucleotide reductase^26^, which might become limiting. While the regulation of pyrimidine homeostasis is complex we may speculate that TbCDA-like is involved in the deamination of cytidine, which leads to uridine that may be converted to uracil via uridine phosphorylase. The further action of uracil phosphoribosyltransferase would result in the formation of UMP and subsequently UTP which is indeed modulated by RNAi and overexpression of TbCDA-like. On the other hand, if deoxycytidine is a substrate it would result in the production of deoxyuridine^27,28^. dCTP hydrolysis to deoxycytidine by the dNTPase TbHD52 and further deamination by TbCDA has been implicated in providing deoxyuridine for phosphorylation to dUMP by thymidine kinase^14^. While TbCDA has an essential role in this process, the additional involvement of TbCDA-like in deoxyuridine production also remains a possibility and could explain the expansion of the dTTP pool in overexpressing cells.

Despite our efforts to produce recombinant TbCDA-like, we have not yet succeeded in identifying its specific substrates. It is noteworthy that canonical orthologues of enzymes with CMP or dCMP deaminase activity have not been identified in trypanosomatids^3^, while a mitochondrial CDA that deaminates cytidine and deoxycytidine, essential for deoxyuridine production in thymidylate biosynthesis, has been well-characterized^27,29^. We hypothesize that TbCDA-like deaminates cytidine derivatives in the cytosol, thus influencing rNTP and dNTP pools. Supporting this notion, the absence of cytidine deaminase in human cells leads to an increase in dCTP and a decrease in dTTP, which impairs replication fork progression^30–32^.

The substantial expansion of the dTTP pool in overexpressing cells may be linked to the increased demand for dNTPs during the DNA damage response, with enhanced deoxyuridine availability promoting *de novo* dTTP synthesis. Imbalances in dNTP pools can compromise genomic stability by affecting DNA replication, repair and recombination fidelity^33–35^. Importantly, the equilibrium between individual dNTPs is critical for genome stability, as skewed dNTP ratios can lead to polymerase errors during DNA synthesis. In human cells, dCTP and dTTP depletion leads to ATR-dependent p53 activation via mismatch repair proteins^36^. Consistent with this, we observed that overexpression of TbCDA-like results in γH2A accumulation, a marker of DNA double-strand breaks and DNA damage signaling in *T. brucei*^16^. This, together with the appearance of a sub-G1 population, supports the notion that TbCDA-like-induced nucleotide imbalances critically affect genomic stability in trypanosomes.

Our localization studies indicate that TbCDA-like is cytosolic and is located in granular structures (possibly P-bodies). Upon serum starvation, it is partially associated with stress granules. It is well established that during starvation conditions in trypanosomes, components from P bodies fuse with other ribonucleoprotein complexes to form mRNA granules, where transcripts are stored These granules are involved in translational repression and mRNA degradation^37^. Hence, while no evidence for nuclear/cytoplasmic mRNA editing exists in *T. brucei* and considering the association with SGs, we could speculate that TbCDA-like deaminates cytidine generated during RNA degradation, which could be recycled for subsequent rounds of mRNA synthesis.

In summary, our findings provide compelling evidence that TbCDA-like plays an important role in maintaining intracellular pyrimidine rNTP and dNTP pools, thereby contributing to nucleotide metabolism in *T. brucei.* Although not essential, perturbations in nucleotide homeostasis resulting from altered TbCDA-like expression lead to DNA damage and cell cycle defects, underscoring its importance in ensuring genomic integrity in trypanosomes.

## Methods

### Generation of cell lines

All *T. b. brucei* cell lines used in this work derived from procyclic forms (*Tb* PF) of line 449 provided by Christine Clayton’s laboratory^38^. Parasites were cultured at 28ºC in SDM-79 media supplemented with 10% fetal bovine serum (FBS) and 7.5 µg·ml^− 1^ hemin.

For RNAi induction, a stem-loop construct was employed. For this purpose, oligonucleotides 5’-AACGGGCCCTAAACCCCATGTCGTCCC-3’ and 5’-GCGGATCCAAGCTTGGTGGTGACCTCGCTG-3’ were used to amplify a 503 bp fragment located between positions 1229 and 1732 of the TbCDA-like coding sequence. The PCR product was first digested with ApaI and HindIII and cloned into the plasmid pGR19^39^ giving rise to the plasmid pGRA10. Cloning of the antisense sequence into pGRA10 digested with HpaI and BamHI, gave rise to plasmid pGRA11.A cell line overexpressing the native protein (*Tb* PF CDA-like-OE) was generated. The oligonucleotides 5’-GCCATATGCCCGTCCATGCCCATG-3’ and 5’-AGATCTCTACTCATGAAGATCCGCC-3’ were used to amplify the coding sequence of TbCDA-like which was subsequently cloned into pGEM-T. The insert released after digestion with NdeI and BamHI was cloned into pAV61^40^ obtaining the plasmid pGRA12 that allows for the expression of TbCDA-like fused to one c-myc at its N-terminus.

Two knock-in (KI) lines were obtained to express from the endogenous locus TbCDA-like fused to 6x c-myc at the N-terminus of the sequence or fused to 12x c-myc at the C-terminus. For the *Tb* PF CDA-like N-terminus (N-ter) KI line, oligonucleotides 5’-CCTAGGATGCCCGTCCATGCCC-3’ and 5’-GGATCCGCCTTGCAAACAGAGC-3’ were used to amplify a 432 bp fragment of the N-terminal region of the TbCDA-like coding sequence (from position 1 to 420). The fragment was cloned into pGEMT to obtain the plasmid pGRA18, which was digested with AvrII and BamHI to release the TbCDA-like fragment that was finally cloned into plasmid pNAT^41^ to express the protein fused to 6x c-myc at the N-terminus. The obtained plasmid, pGRA21, was linearized by digestion with KpnI for subsequent transfection of *Tb* PFs allowing homologous recombination into the target locus, when introduced by electroporation.

In the case of the *Tb* PF CDA-like C-terminus (C-ter) KI line, oligonucleotides 5’-AAGCTTTGGTGACCTCGCTGATGACG-3’ and 5’-TCTAGACTCATGAAGATCCGCCAC-3’ were used to amplify a 536 bp fragment of the C-terminal region of the TbCDA-like coding sequence (positions 1241 to 1764) which was cloned into pGEMT to obtain the plasmid pGRA19. Restriction enzymes HindIII and XbaI were used to digest pGRA19 and the released fragment was cloned into pNAT to express the protein fused to 12x c-myc at the C-terminus. The resulting plasmid, pGRA20, was digested with BlpI and used to transfect *Tb* PFs allowing homologous recombination into the target locus, when introduced by electroporation. In Suplementary Fig. S3 the schematics of all the constructs are illustrated.

### *T. brucei* transfection

Transfections were carried out by electroporation in Zimmerman buffer with 10 µg of NotI-linearized plasmid DNA as previously described^42^. The selection drugs used were: puromycin 1 µg·ml^− 1^, hygromycin 50 µg·ml^− 1^ or blasticidin 10 µg·ml^− 1^. RNAi and TbCDA-like overexpression were induced with doxycycline 1 µg·ml^− 1^.

### Antibody generation

Oligonucleotides 5’-GCCATATGCCCGTCCATGCCCATG-3’ and 5’-GCAAGCTTCTACTCATGAAGATCCGCC-3’ were used to amplify the TbCDA-like coding sequence from *T. brucei* genomic DNA. The PCR product was cloned into pGEMT and subsequently digested with HindIII and NdeI to release the fragment with the coding sequence for TbCDA-like which was cloned into pET28a to obtain pGRA5. *E. coli* BL21 (DE3) cells were transformed with pGRA5 to express TbCDA-like fused to a 6x His tail at the N-terminus (His-TbCDA-like).

Expression was induced for subsequent purification under denaturing conditions to obtain a polyclonal antibody since the recombinant protein was not detected in the soluble fraction. Specifically, *E. coli* BL21 (DE3) cells transformed with pGRA5 were grown in 100 ml of LB medium with kanamycin 50 µg·ml^− 1^ at 37ºC and shaking overnight. The next day, the culture was completed to a final volume of 2 L of LB with kanamycin (50 µg·ml^− 1^) and 1 mM IPTG was added when the culture reached an OD600 value of 0.6. After 5 h of shaking at 37 °C, the cells were collected and stored at -80ºC until use. The bacterial pellet obtained was resuspended in 50 ml of lysis buffer (50 mM Tris-HCl, 20 mM imidazole, 8 M urea, 0.5 M NaCl, pH 7.5, cocktail of protease inhibitors (complete mini, EDTA-free protease inhibitor cocktail tablets; Roche) and sonicated. The whole extract was incubated for 2 h at room temperature with Ni Sepharose High Performance affinity media (GE Healthcare), previously equilibrated with lysis buffer. The resin was then washed with lysis buffer and washing buffer (50 mM Tris-HCl, 50 mM imidazole, 8 M urea, 0.5 M NaCl, pH 7.5, cocktail of protease inhibitors) and the protein was eluted using 50 mM Tris-HCl, 500 mM imidazole, 8 M urea and 0.5 M NaCl, pH 7.5 containing protease inhibitors.

The protein was purified by electroelution using the Protean II Xi Cell System (Bio-Rad) and performing the separation in a 10% acrylamide gel at 200 V for 5 h. The bands corresponding to TbCDA-like were cut from the gel and the protein was isolated using an Electro-Eluter 422 system (Bio-Rad) at 30 mA for 5 h at room temperature. The fractions obtained were analyzed by SDS-PAGE and the Bradford method was used to quantify the protein concentration.

To obtain a polyclonal antibody against TbCDA-like, a single rabbit was immunized with purified TbCDA-like. Four inoculations were carried out, each of them with ~ 400 µg of protein in 0.5 ml of PBS (137 mM NaCl, 2.7 mM KCl, 1.8 mM KH_2_PO_4_, 10 mM Na_2_HPO_4_, pH 7.4) and Freund’s Adjuvant (Sigma) (1:1 ratio). In order to isolate IgGs, the serum was purified by affinity chromatography using a protein A-binding resin (protein A sepharose CL-4B, GE Healthcare) following the manufacturer’s instructions.

### Generation of *T. brucei* extracts and Western blot

5 × 10^6^ parasites per sample were collected by centrifugation, washed with PBS, resuspended in buffer containing urea (6 M urea, 10 mM Na_2_HPO_4_, 1% β-mercaptoethanol, 1% SDS, pH 7) and boiled after the addition of loading sample buffer (67.5 mM Tris-HCl, pH 6.8, 3% SDS, 10% glycerol, 5% β-mercaptoethanol). The extracts obtained were subjected to SDS-PAGE analysis and proteins were transferred to a polyvinylidene difluoride membrane (PVDF, GE Healthcare) and incubated with rabbit polyclonal anti-TbCDA-like (1:2500) or mouse monoclonal anti-β-tubulin (Sigma) (1:5000) antibodies. Bound antibodies were revealed by using goat anti-rabbit IgG (1:5000) or goat anti-mouse IgG (1:3000) antibodies (Promega) by using the ECL Select kit (GE Healthcare).

### Immunofluorescence analysis

To determine the intracellular localization of TbCDA-like, immunofluorescence studies were performed. Log-phase parasites were harvested and subsequently incubated in 1 ml of culture medium with 100 µM MitoTracker Red CMXRos (Invitrogen) for 15 min at 28ºC. Fixation was carried out on Poly-L-lysine-coated slides for 20 min with 4% p-formaldehyde in wash solution (1X PBS, 0.2% Tween 20). Subsequently, the slides were washed twice and then the sample was permeabilized for 75 min at room temperature with 1% NP-40 in blocking solution (1% blocking reagent (Roche) in wash solution). After washing once, incubation with rabbit polyclonal anti-TbCDA-like (1:25) or mouse monoclonal anti-c-myc (1:100) (Sigma) in blocking solution was carried out. Alexa Fluor 488 goat anti-rabbit IgG (1:40) or goat anti-mouse IgG (1:40) (Sigma) were used as secondary antibodies.Samples were dehydrated in methanol for 1 min and mounted using ProLong Gold Antifade Mountant with DAPI (Invitrogen).

To determine whether TbCDA-like relocalizes to stress granules under nutritional stress conditions, the *Tb* PF CDA-like N-ter KI line was used. 2.5 × 10^7^ cells were harvested in log-phase, washed and maintained in culture for 60, 120–180 min in PBS. After the indicated time, immunofluorescence was performed as previously described. Rabbit polyclonal anti-TbDhh1 (1:100), anti-TbDRBD3 (1:100), anti-TbScd6 (1:100) or mouse monoclonal anti-c-myc (1:100) were used as primary antibodies and Alexa Fluor 594 goat anti-rabbit IgG and Alexa Fluor 488 goat anti-mouse IgG as secondary antibodies.

Vertical stacks of 30–40 slices (0.2 μm steps) per image were captured with an Olympus wide-field microscope (100X objective) using Cell-R IX81 software. Huygens Essential software (version 3.3; Scientific Volume Imaging) was used for image deconvolution and Fiji software (version 1.5e; ImageJ) was used for analysis. The degree of colocalization was determined by Pearson’s correlation coefficient calculated with the JACoP plugin^43^ of Fiji/ImageJ software in at least 10 cells per condition. 3D construction images were obtained with the three-dimensional reconstruction module of Leica Application Suite X software.

Regarding the study of the area and number of granules per cell, Fiji/ImageJ software was used with a minimum particle size of 0.05 µm^2^. The analysis was performed on the z-projection of the images obtained by the layer summation method. 10–22 cells per condition were analyzed.

For genomic DNA damage quantification studies in *Tb* PFs, a polyclonal anti-γH2A antibody (1:500) followed by an anti-rabbit IgG conjugated with Alexa Fluor 488 (1:100) were used^16^. Sample preparation was performed as described above and a Zeiss Axio Imager A1 microscope was used for image acquisition and analyzed using Fiji software (version 1.5e; ImageJ).

To discard the colocalization of TbCDA-like with glycosomes the antibody anti-LmMVAK was used as a glycosomal marker. Images were obtained for the N-ter KI line. An anti-c-myc monoclonal antibody (1:100) and anti-LmMVAK polyclonal antibody (1:2400) were used as primary antibodies. Anti-mouse IgG and anti-rabbit IgG antibodies conjugated with Alexa Fluor 488 and Alexa Fluor 594 fluorochromes, respectively, were used as secondary antibodies. Nuclei and kinetoplasts were labelled with DAPI. Images were obtained on a Leica DMi8 microscope (100X objective) and deconvoluted with Huygens Essential software (version 3.3; Scientific Volume Imaging). Images were analyzed with Fiji/ImageJ software.

### Fluorescence-activated cell sorting (FACS) analysis and quantification of nuclei and kinetoplasts

Cell cycle analysis by flow cytometry was performed on *Tb* PF, *Tb* PF CDA-like-RNAi and *Tb* PF CDA-like-OE lines. In each case, 1 × 10^7^ cells were harvested and the pellet was washed twice with PBS. Parasites were fixed overnight at -20 °C in a solution of 70% ethanol and PBS and subsequently treated with 40 µg·ml^− 1^ propidium iodide and RNase A 10 µg·ml^− 1^ in PBS for 30 min at 37ºC. Fluorescence was measured with a FACScalibur cytometer (Becton Dickinson) and cell populations analyzed using the FlowJo version 7.6 software.

The cell cycle was also studied by DAPI staining to analyze the number and morphology of nuclei and kinetoplasts by fluorescence microscopy. 1 × 10^6^ parasites were collected and fixed for 20 min with 4% p-formaldehyde and mounted with ProLong Gold Antifade Mountant with DAPI (Invitrogen). A Zeiss Axio Imager A1 microscope was used for image acquisition.

### Metabolomic analysis

Metabolomic studies were performed by Metabolon Inc. on the *Tb* PF CDA-like-RNAi line after 6 days of induction with doxycycline and compared with the *Tb* PF parental line (See Supplementary Spreadsheet S1). Four biological replicates were tested and, in each case, 1 × 10^9^ cells were harvested, washed and immediately frozen in liquid nitrogen. 565 different metabolites were identified. Briefly, the samples were prepared according to the MicroLab STAR system and analyzed by various mass spectrometry methods, namely: reversed-phase ultra-high resolution liquid chromatography coupled to tandem mass spectrometry (RP/UPLC-MS/MS) with positive or negative ion mode electrospray ionization (ESI) and ultra-high resolution hydrophilic interaction chromatography coupled to tandem mass spectrometry (HILIC/UPLC-MS/MS) with negative ion mode ESI.

### Measurement of intracellular nucleotides

Determination of ribonucleoside triphosphates (rNTPs) was performed by anion exchange chromatography on an HPLC system (Pharmacia). 1 × 10^8^ cells were collected for each condition. After washing with PBS, the pellet was resuspended in a solution of water and 0.4 N perchloric acid 50% (v/v), incubated for 5 min on ice and centrifuged for 10 min at 16,000 x *g*. Finally, the supernatant was added to 125 µl of 0.5 M ammonium phosphate buffer pH 3.4 and the sample was loaded onto a Partisphere 5 μm SAX column (Hichrom). Separation of the different rNTPs took place under isocratic elution conditions with 0.5 M ammonium phosphate buffer pH 3.4. For nucleotide quantification, a standard curve was used for each rNTP.

The determination of deoxyribonucleoside triphosphates (dNTPs) was performed by the DNA polymerase assay^12^, based on the incorporation of tritium^3^H)-labelled dATP into sequence-specific oligonucleotides: for dCTP 5’-TTTGTTTGTTTGTTTGTTTGGGCGGTGGAGGCGG-3’ and for dTTP and dUTP measurements, 5’-TTTATTTATTTATTTATTTAGGCGGTGGAGGCGG-3’ were used. The oligonucleotide 5’-CCGCCTCCACCGCC-3’ was used as primer.

2 × 10^6^ parasites were collected per sample, washed with PBS and incubated in 250 µl of 60% methanol at -20 °C overnight. The samples were boiled 5 min and centrifuged for 20 min at 17,000 x *g* and 4ºC. The supernatant was transferred to a fresh tube and vacuum-dried in a SpeedVac DNA 120 (Savant) and then resuspended in a final volume of 100 µl reaction buffer with the appropriate oligonucleotide containing the DNA primer (32 nM), NE Buffer 2 (New England Biolabs), 0.3 units of the Klenow fragment of DNA polymerase I (New England Biolabs) and 0.025 nmol of^3^H]-dATP (PerkinElmer). The reaction mixture was incubated for 15 min at 25 °C and the reaction was stopped by adding 10 mM EDTA and heating 20 min at 75 °C. DNA was precipitated with 10% (v/v) TCA for 30 min at 4ºC. Samples were filtered with GF/C glass microfiber filters (Whatman) that were washed with 30 ml of 5% TCA and dried with 3 ml of ethanol. Radioactivity was determined with a LS 6500 Multi-Purpose scintillation counter (Beckman Coulter). For nucleotide quantification, a standard curve was used for each dNTP.

## Electronic supplementary material

Below is the link to the electronic supplementary material.

Supplementary Material 1

Supplementary Material 2

## Data Availability

The datasets generated during and/or analyzed during the current study are available from the corresponding author on reasonable request.
