# Correction: A cytidine deaminase-like protein modulates pyrimidine nucleotide homeostasis in *Trypanosoma brucei*

**DOI:** 10.1038/s41598-025-10023-z

**Published:** 2025-07-08

**Authors:** Ana Moro-Bulnes, Cristina Bosch-Navarrete, Pablo Antequera-Parrilla, Santiago Castanys, Antonio E. Vidal, Luis Miguel Ruiz-Pérez, Guiomar Pérez-Moreno, Dolores González-Pacanowska

**Affiliations:** Instituto de Parasitología y Biomedicina “López-Neyra” (IPBLN), CSIC, Parque Tecnológico de Ciencias de la Salud, Avda. del Conocimiento, 17, Armilla, Granada, 18016 Spain

Correction to: *Scientific Reports* 10.1038/s41598-025-00942-2, published online 09 May 2025

In the original version of this Article, the names of Guiomar Pérez-Moreno and Dolores González-Pacanowska were inadvertently duplicated in the author list.

The original Article has been corrected.

