# Supplementary material for: A cytidine deaminase-like protein modulates pyrimidine nucleotide homeostasis in Trypanosoma brucei
Source: Sci Rep. 2025 May 9;15:16160. doi: 10.1038/s41598-025-00942-2 (PMC12064671; doi:10.1038/s41598-025-00942-2)
Supplement: Supplementary file 2 — Supplementary Material 2 [file 41598_2025_942_MOESM2_ESM.pdf]

SUPPLEMENTARY INFORMATION

A cytidine deaminase-like protein modulates pyrimidine nucleotide homeostasis in *Trypanosoma brucei*

Ana Moro-Bulnes[a], Cristina Bosch-Navarrete[a], Pablo Antequera-Parrilla[a], Santiago Castanys[a], Antonio E. Vidal[a], Luis Miguel Ruiz-Pérez[a], Guiomar Pérez-Moreno[a]* and Dolores González-Pacanowska[a]*.

[a] *Instituto de Parasitología y Biomedicina "López-Neyra" (IPBLN), CSIC, Parque Tecnológico de Ciencias de la Salud. Avda. del Conocimiento, 17. 18016 Armilla (Granada), Spain.*

** Co-corresponding authors. Email: dgonzalez@ipb.csic.es (Dolores González Pacanowska) and guiomar@ipb.csic.es (Guiomar Pérez Moreno)*

FIGURE S1

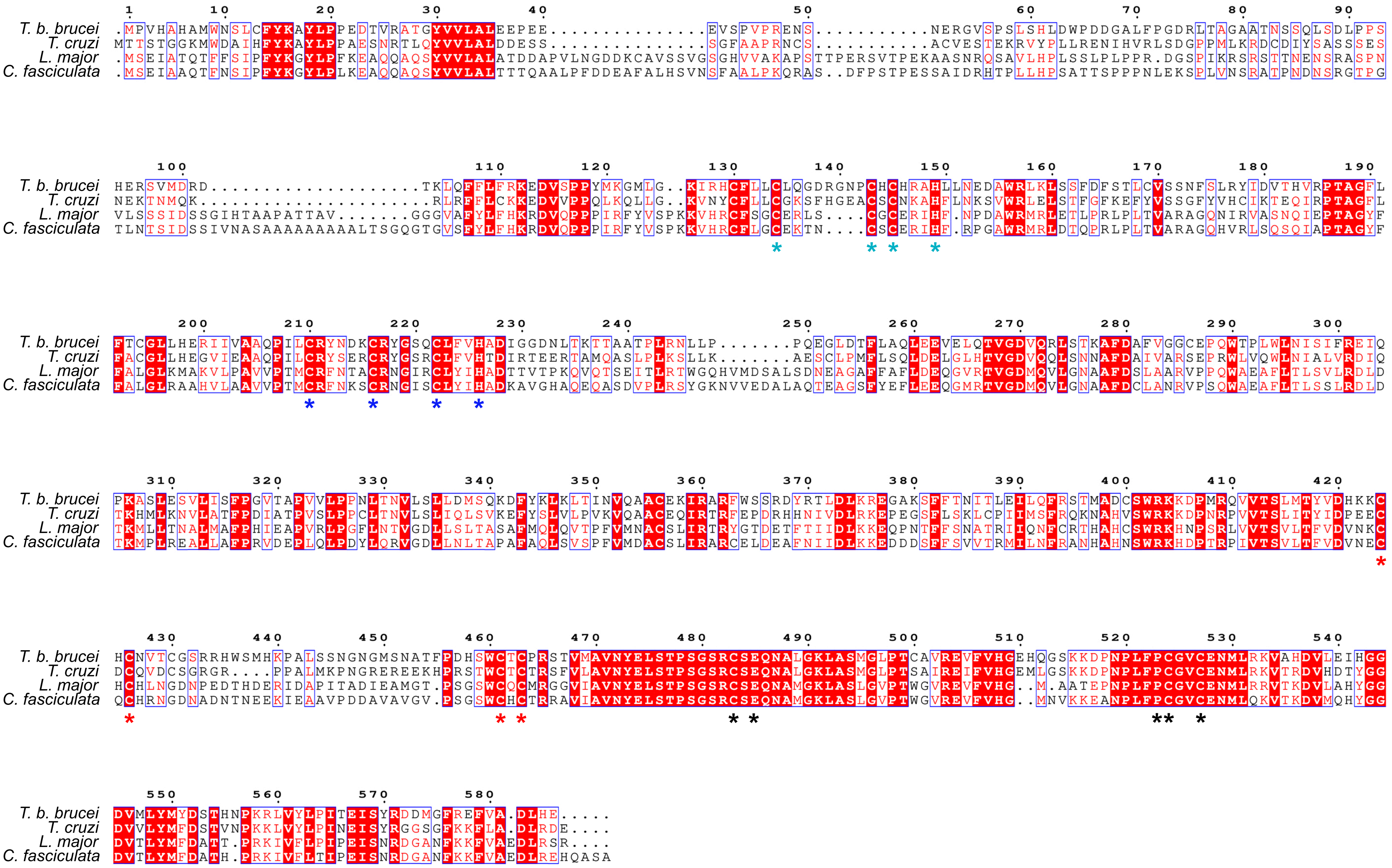

Supplementary Fig. S1. Sequence alignment of CDA-like orthologs of kinetoplastids. The sequences were aligned using MultAlin and visualized with ESPript. Columns showing the conservation of residues are color-coded according to the level of identity between the sequences. *T. b. brucei* (Tb927.10.8850), *Trypanosoma cruzi* (TcCLB.511039.39), *Leishmania major* (LmjF.36.5940), *Crithidia fasciculata* (CFAC1_280067400). Accession numbers from TriTrypDB. The residues in the two CCCH domains zinc finger are indicated by cyan and blue asterisks. Cysteines that form the C2C2 domain, potentially responsible for zinc coordination, are marked with red asterisks. Amino acids constituting the catalytic core of the deaminase domain are highlighted with black asterisks.

FIGURE S2

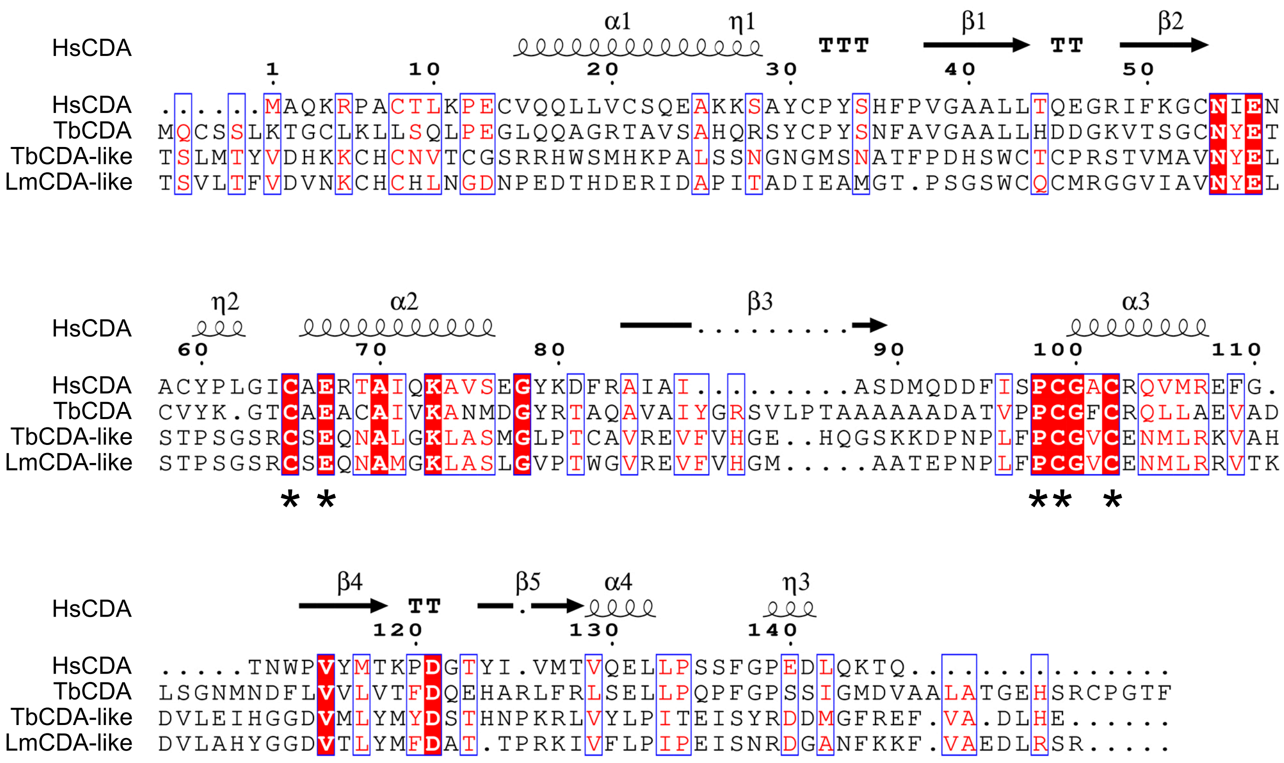

Supplementary Fig. S2. Alignment of two cytidine deaminase sequences (HsCDA and TbCDA) with the deaminase domain-containing region of TbCDA-like and LmCDA-like. Amino acids forming the catalytic core of the deaminase domain are highlighted with black asterisks. *Homo sapiens* CDA (NCBI ID: N_001776.1), *T. b. brucei* CDA (TriTrypDB ID: Tb927.9.3000), *T. b. brucei* CDA-like (TriTrypDB ID: Tb927.10.8850), *Leishmania major* CDA-like (TriTrypDB ID: LmjF.36.5940).

FIGURE S3

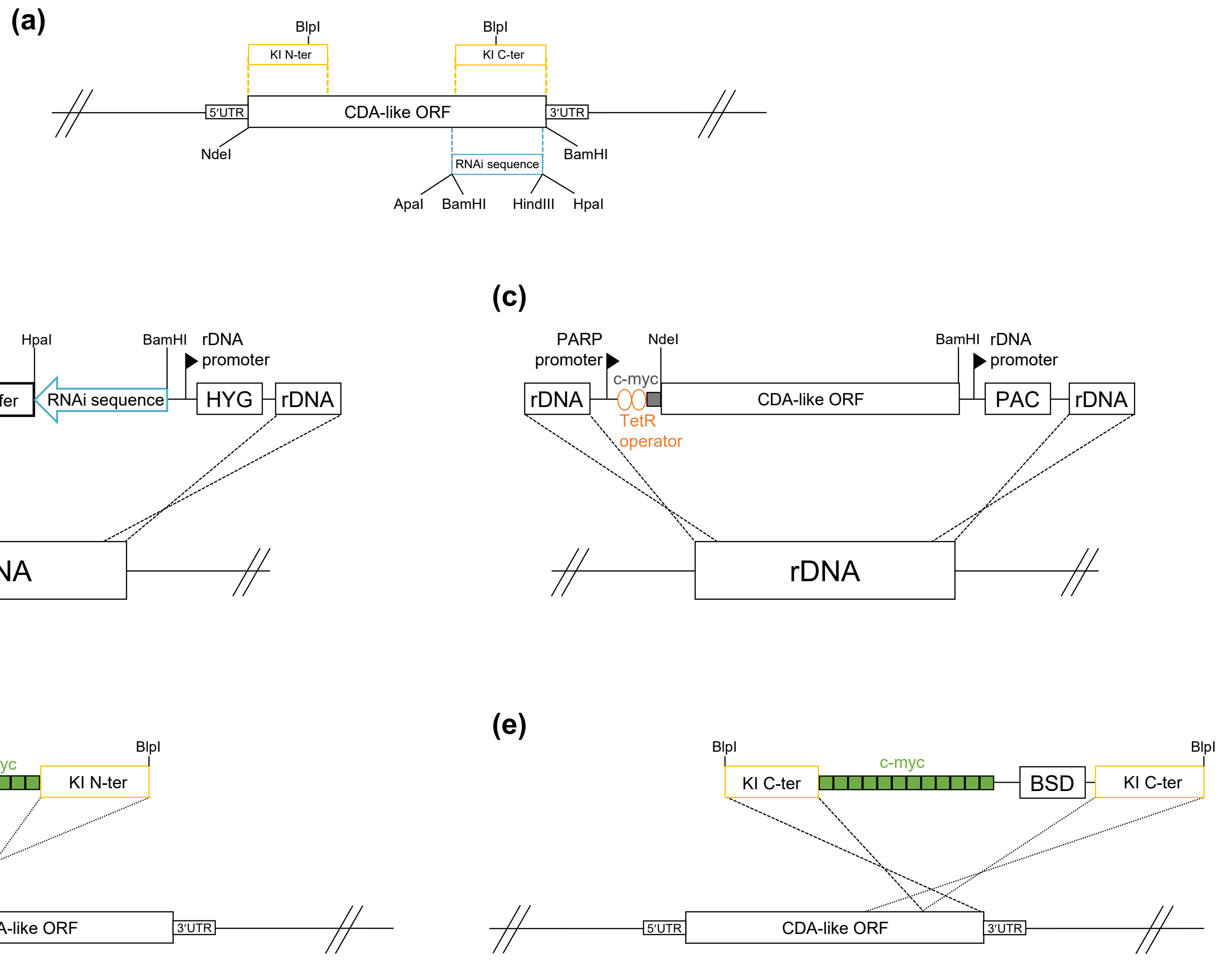

Supplementary Fig. S3. (a) Schematic representation of the *CDA-like* gene and the regions selected for different constructs. (b, c) RNAi and OE constructs and their integration into the *rDNA* locus. (d, e) Schematic representation of the N-terminal (KI N-ter) and C-terminal (KI C-ter) knock-in constructs, respectively, and their integration into the *CDA-like* locus.

FIGURE S4

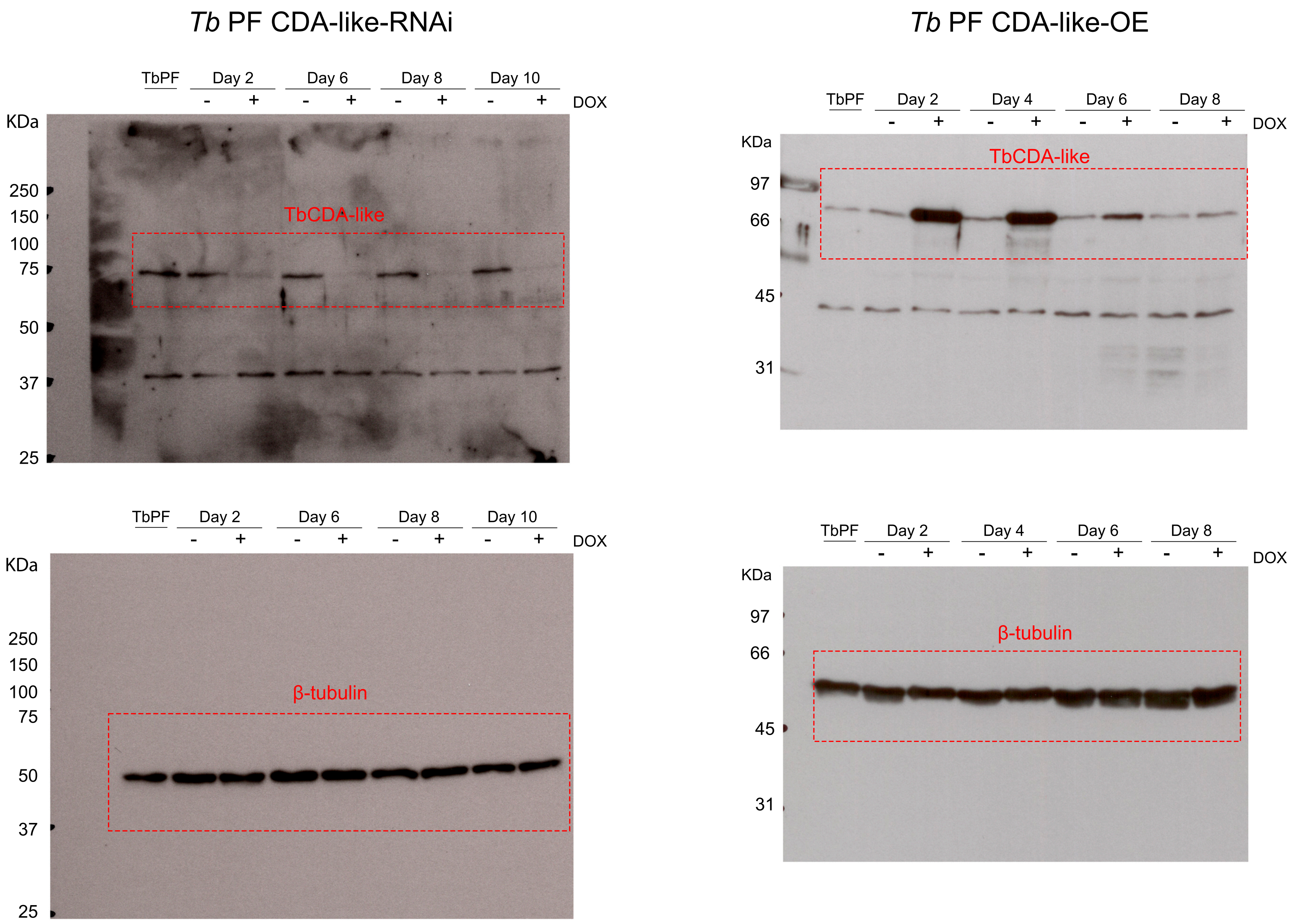

Supplementary Fig. S4. Full size images of Western blots presented in Fig. 1b and d. The regions shown in Fig. 1 are delineated with a red dashed line. Left upper panel: Western blot showing TbCDA-like protein levels in whole cell extracts from parasites upon RNAi-mediated depletion. Left lower panel: β-tubulin was used as loading control. Right upper panel: Western blot showing TbCDA-like protein levels in whole cell extracts from overexpressing parasites. Right lower panel: β-tubulin was used as loading control.

FIGURE S5

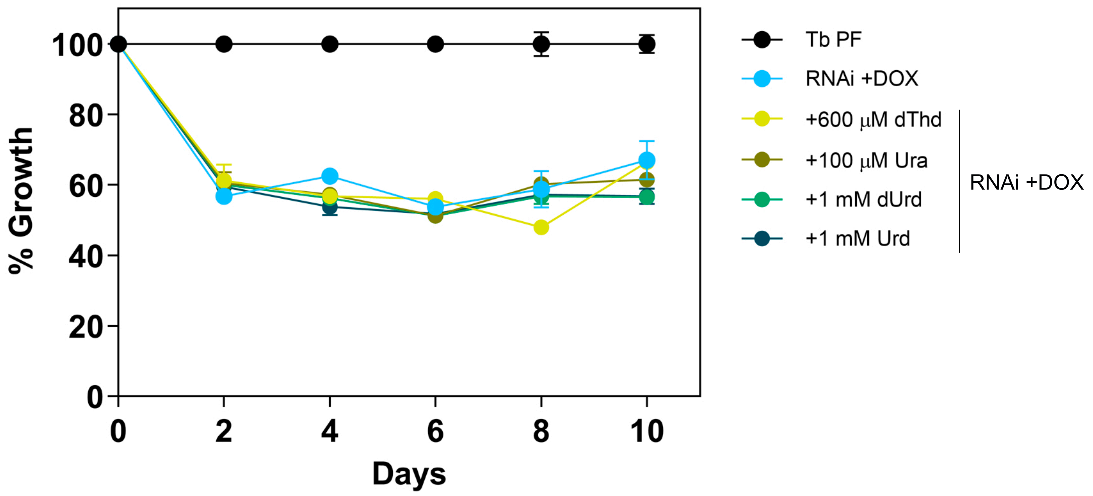

Supplementary Fig. S5. Effect of supplementation with different nucleosides and pyrimidine bases on TbCDA-like RNAi. Growth curve of the parental (*Tb* PF) and RNAi cell lines (*Tb* PF CDA-like-RNAi) cultured with doxycycline (+DOX) in the presence of different concentrations of thymidine (dThd), uracil (Ura), deoxyuridine (dUrd) or uridine (Urd).

FIGURE S6

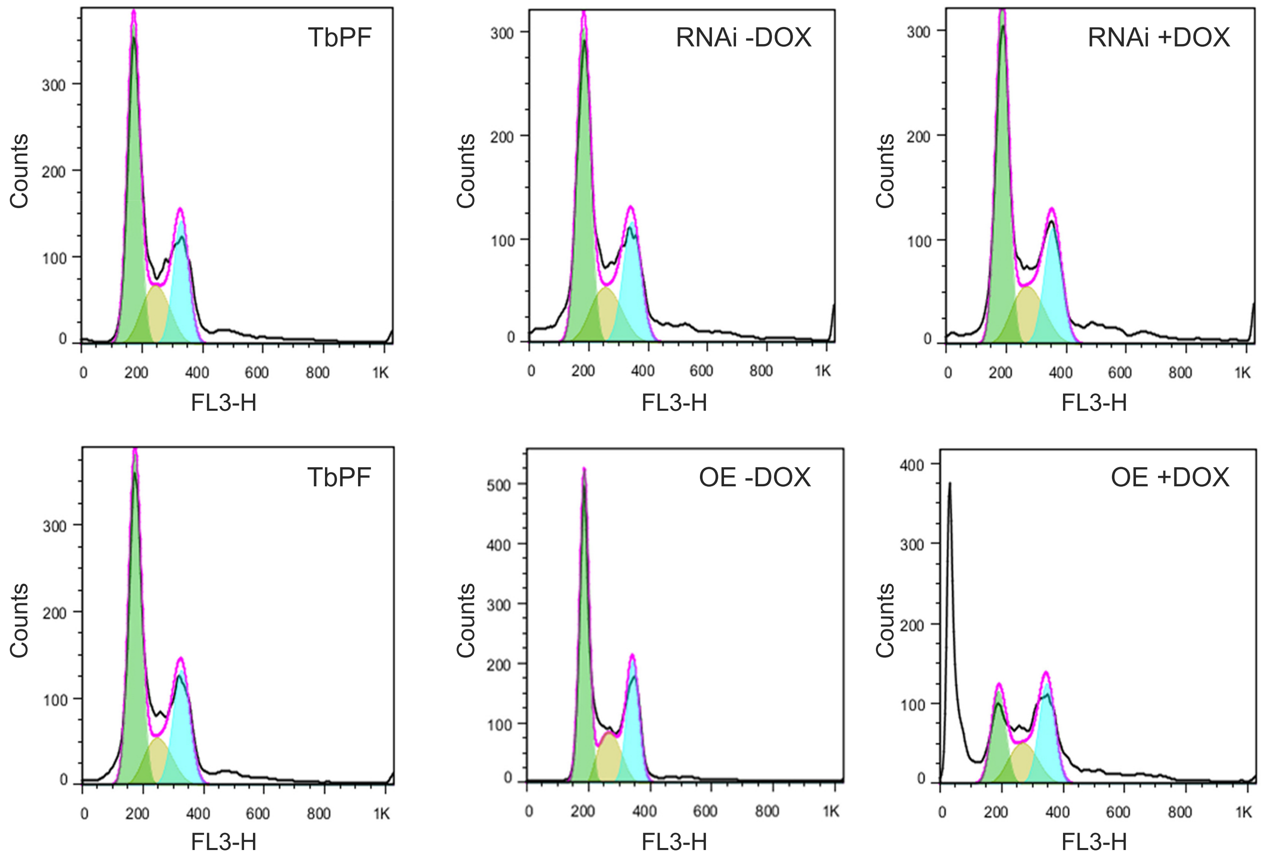

Supplementary Fig. S6. Representative flow cytometry histograms of cell cycle analysis of *Tb* PF, *Tb* PF CDA-like-RNAi and *Tb* PF CDA-like-OE cell lines. Green, ocher and blue shading represent cell populations for G1, S and G2/M phases, respectively.

FIGURE S7

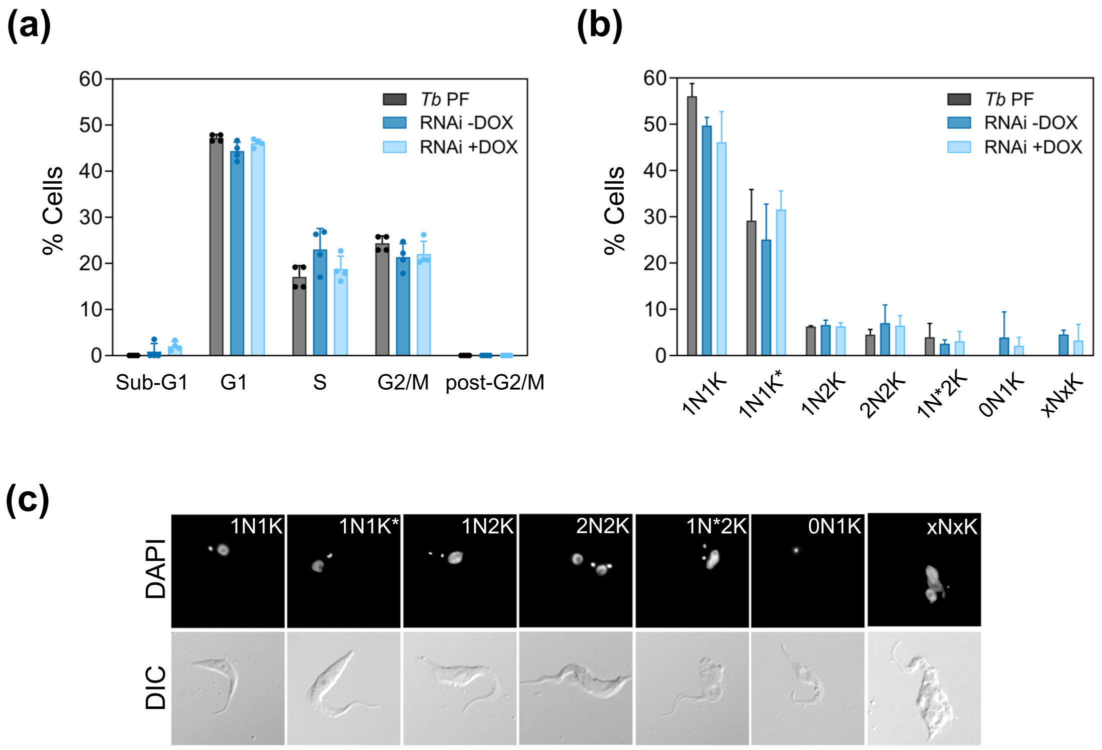

Supplementary Fig. S7. Impact of TbCDA-like RNAi induction on cell cycle progression. (a) Cell cycle progression analyzed by FACS after 2 days of Tb CDA-like-RNAi induction. (b) Quantification of nuclei (N) and kinetoplasts (K) by DAPI staining after 2 days of RNAi induction N* and K* denote elongated nuclei and kinetoplasts, respectively, undergoing mitosis or kinetoplast division. Data represent the mean ±SD of approximately 300 cells from 3 independent experiments. (c) Representative images obtained with the Zeiss Axio Imager A1 fluorescence microscope after DAPI staining, corresponding to the different populations.

FIGURE S8

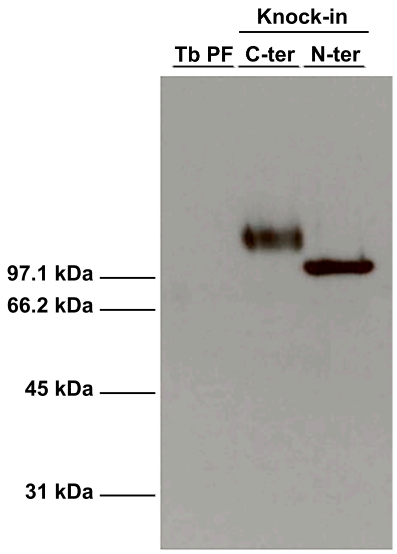

Supplementary Fig. S8. The anti-c-myc antibody specifically recognized the c-myc epitope-tagged TbCDA-like in both *Tb* PF C-ter KI and *Tb* PF N-ter KI cell lines. Parental *Tb* PF was used as negative control.

FIGURE S9

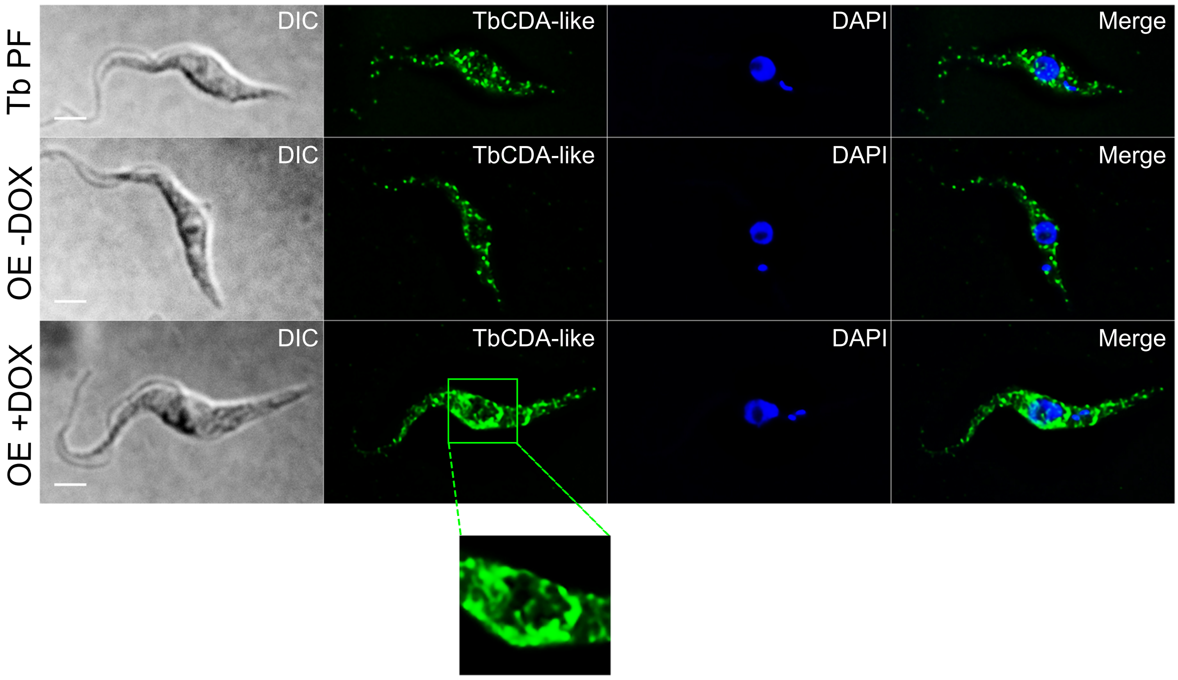

Supplementary Fig. S9. The images show the cytosolic localization of TbCDA-like in the parental line (*Tb* PF) as well as in *Tb* PF overexpressing TbCDA-like (*Tb* PF CDA-like-OE) without or with DOX. Immunofluorescence was performed after 2 days of induction of TbCDA-like. A rabbit polyclonal anti-TbCDA-like and monoclonal anti-IgG antibody conjugated with Alexa Fluor 488 fluorochrome were used. Nuclei and kinetoplasts were labelled with DAPI. In both cases images were obtained on an Olympus IX81 microscope and deconvoluted with Huygens Essen-

Fiji/ImageJ software. Bar, 10 µm.

FIGURE S10

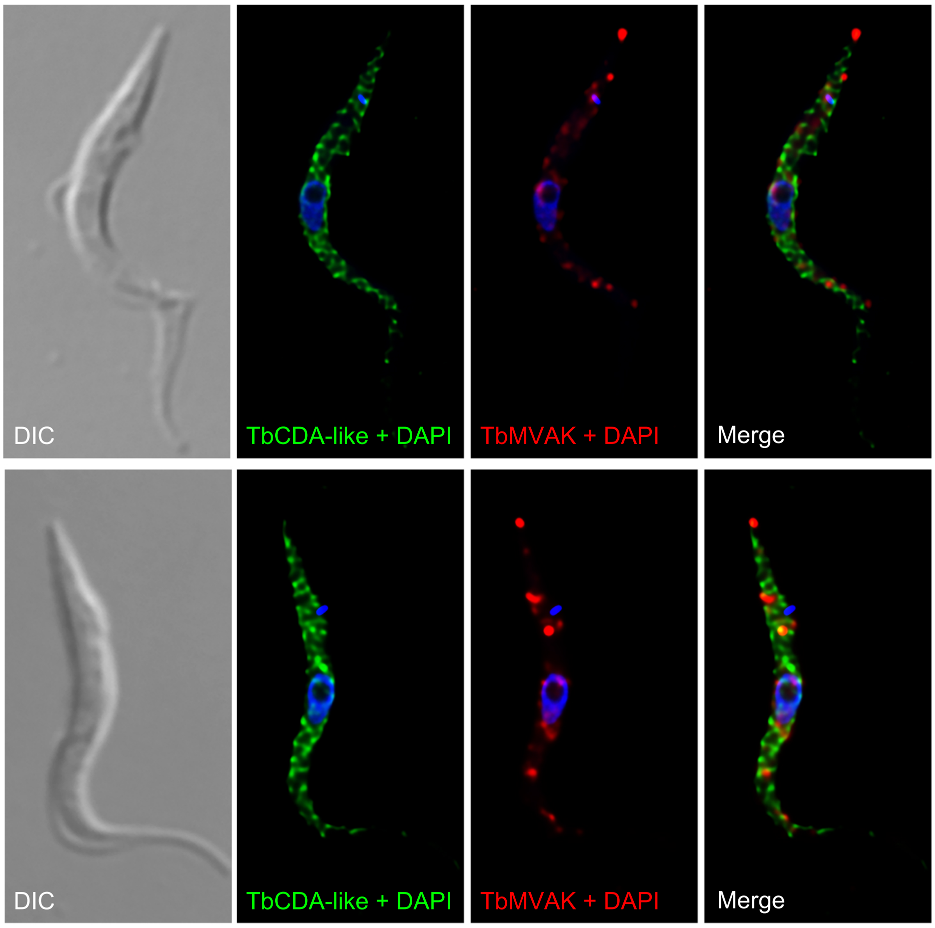

Supplementary Fig. S10. Immunofluorescence images showing that TbCDA-like does not localize to the glycosome. LmMVAK was used as a glycosomal marker (red). Images were obtained for the KI line for TbCDA-like fused to 6xc-myc at the N-terminus. An anti-c-myc monoclonal antibody and anti-LmMVAK polyclonal antibody were used as primary antibodies. Anti-mouse IgG and anti-rabbit IgG antibodies conjugated with Alexa Fluor 488 and Alexa Fluor 594 fluorochromes, respectively, were used as secondary antibodies. Nuclei and kinetoplasts were labelled with DAPI. Images were obtained on a Leica DMi8 microscope and deconvoluted with Huygens Essential software (version 3.3; Scientific Volume Imaging). Images were analyzed with Fiji/ImageJ software.
